# Priming by Hexanoic Acid Induce Activation of Mevalonic and Linolenic Pathways and Promotes the Emission of Plant Volatiles

**DOI:** 10.3389/fpls.2016.00495

**Published:** 2016-04-12

**Authors:** Eugenio Llorens, Gemma Camañes, Leonor Lapeña, Pilar García-Agustín

**Affiliations:** Grupo de Bioquímica y Biotecnología, Departamento de Ciencias Agrarias y del Medio Natural, Universitat Jaume ICastellón, Spain

**Keywords:** induced resistance, citrus, *Alternaria alternata*, volatiles, non-targeted metabolomics

## Abstract

Hexanoic acid (Hx) is a short natural monocarboxylic acid present in some fruits and plants. Previous studies reported that soil drench application of this acid induces effective resistance in tomato plants against *Botrytis cinerea* and *Pseudomonas syringae* and in citrus against *Alternaria alternata* and *Xanthomonas citri.* In this work, we performed an in deep study of the metabolic changes produced in citrus by the application of Hx in response to the challenge pathogen *A. alternata*, focusing on the response of the plant. Moreover, we used ^13^C labeled hexanoic to analyze its behavior inside the plants. Finally, we studied the volatile emission of the treated plants after the challenge inoculation. Drench application of ^13^C labeled hexanoic demonstrated that this molecule stays in the roots and is not mobilized to the leaves, suggesting long distance induction of resistance. Moreover, the study of the metabolic profile showed an alteration of more than 200 molecules differentially induced by the application of the compound and the inoculation with the fungus. Bioinformatics analysis of data showed that most of these altered molecules could be related with the mevalonic and linolenic pathways suggesting the implication of these pathways in the induced resistance mediated by Hx. Finally, the application of this compound showed an enhancement of the emission of 17 volatile metabolites. Taken together, this study indicates that after the application of Hx this compound remains in the roots, provoking molecular changes that may trigger the defensive response in the rest of the plant mediated by changes in the mevalonic and linolenic pathways and enhancing the emission of volatile compounds, suggesting for the first time the implication of mevalonic pathway in response to hexanoic application.

## Introduction

Plants are static organisms that have been forced to develop mechanisms to adapt and survive to different stresses and unfavorable environments. To face the enormous number of microorganisms that could hurt them, they arm themselves with molecular shields against their attackers. Innate resistance of plants is based on constitutive physical and chemical barriers that are able to avoid the infection of a large number of challenge pathogens. When plants are attacked, in addition of the constitutive barriers, batteries of inducible defenses are activated to interfere with the colonization. These are the result of a complex signaling network, in which the hormones jasmonic acid (JA), salicylic acid (SA), and ethylene (ET) are involved (Pieterse et al., 2012). This innate immunity can be stimulated, achieving an enhanced level of resistance and leading the phenomenon known as induced resistance (IR). This term describes the state of resistance in plants triggered by biological or chemical agents, which protects non-exposed plant parts against a future attack by pathogenic microbes or herbivorous insects. Thus, resistance is expressed not only locally at the site of induction, but also systemically in other unexposed parts. Depending on the kind of the pathogen and the subsequent response of the plant, this IR can be divided in systemic acquired resistance (SAR), herbivore-induced resistance (HIR), and induced systemic resistance (ISR) (Pieterse et al., 2014). Besides these mechanisms, plants are able to release volatile compounds that also play a role in the control of defense, preparing neighbor plants for an enhanced response upon subsequent attack (Farmer, 2001; Erb et al., 2015). In addition, release of volatile compounds is a self-defense system by inducing defenses in the same plant, preparing adjacent leaves that are not directly connected via the plant’s vascular system (Heil and Ton, 2008).

Plants also have the ability to retain memory of pathogenic attacks, phenomenon known as priming, which is usually leads to a faster and stronger response of the basal defenses against a subsequent stress (Achuo et al., 2004; Conrath, 2009; Jung et al., 2009). Moreover, this state of priming in plants can be induced by the application of natural and chemical compounds (Conrath, 2009; Gozzo and Faoro, 2013; Yu et al., 2014). Primed plants can show a wide variety of responses such as the control of stomata closure (Jakab et al., 2005), generation of reactive oxygen species (Pastor et al., 2013), changes in the hormonal homeostasis (Conrath, 2009; Aranega Bou et al., 2014), and emission of plant volatiles (Winter et al., 2012).

In the lasts years, several carboxylic acids have been widely studied as priming inducers (Flors et al., 2001, 2003; Vicedo et al., 2006; Gamir et al., 2012). Hexanoic acid (Hx) is a monocarboxylic acid that can induce plant defense responses when is used as a priming agent. Several studies have demonstrated a wide range of activity for this compound, displaying protection against fungal or bacterial diseases in different crops (Aranega Bou et al., 2014). These studies revealed that hexanoic acid-induced resistance (Hx-IR) protects plants against fungal diseases by priming callose deposition and enhancement of JA-dependent defenses, as well as accumulation of phenolic compounds in long lasting defense (Vicedo et al., 2009; Llorens et al., 2015a). Moreover, the basal resistance and Hx-IR in plants against bacterial diseases was recently analyzed. Tomato plants inoculated with *Pseudomonas syringae* showed and induction of SA and increased the accumulation of 12-oxo-phytodienoic acid (OPDA) (Scalschi et al., 2013). On the other hand, application of Hx in citrus against *Xanthomonas citri* induced the expression of PR genes and callose accumulation (Llorens et al., 2015b). However, the changes induced by the application of this molecule and the mechanisms that underlay the hexanoic RT are still unclear.

The use of new chromatographic techniques to study the metabolomics changes in RT and plant–microbe interactions has allowed unraveling the implication of certain metabolites in these processes. In this way, several secondary metabolites have recently been identified as possible players in plant priming such as azelaic acid (Jung et al., 2009), or pipecolic acid (Návarová et al., 2012). The combination of non-targeted and targeted quantitative analysis allows us the possibility to perform a deep study of the alterations produced by both, the priming agent and the pathogen response. The characterization of the elements participating in plant defense, the targets and changes produced by the priming agent allows us to develop more accurate systems to protect the plants against different stresses. For this reason, in this work we studied the movement of Hx in the plant after soil drench application using isotope labeled Hx. We also determined the metabolic profile of treated and treated and infected plants, which demonstrates the implication of, at least, two different defensive pathways. Finally, we corroborated the implication of these two pathways by the analysis of volatile compound emission and marker genes expression.

## Materials and Methods

### Plant Material and Pathogen Inoculation

For ^13^C assay, 4 months plantlets of citrange Carrizo (Beniplant, Valencia, Spain) with a single shoot were used.

For inoculation, metabolomics and volatile emission assays, we used 2-year-old Fortune mandarin plants (*Citrus clementina* hort. ex Tanaka x Dancy mandarin) grafted onto Carrizo citrange plants (*Citrus sinensis* L. Osbeck x *Poncirus trifoliata* Blanco) and grown in a greenhouse in 10-L pots using peat as substrate (Vivercitrus, Alcanar, Spain). One month before the commencement of each experiment, the leaves were removed to encourage uniform sprouting. The leaves with a size that was suitable for inoculation (75% expanded) were labeled and infected.

Spores of *Alternaria alternata* were collected from 10- to 15-day-old cultures with sterile water containing 0.02% (v/v) Tween-20. The solutions were filtered, quantified with a hemocytometer and adjusted to 10^5^ spores/mL. Infection was carried out by dispensing 5 μL of the spore solution onto each leaf. Ninety six hour after inoculation, the diameter of necrosis and the number of infected leaves was recorded. Leaves located immediately next to the inoculation point on the same shoot were used. Entire fresh leaves were used for the volatile emission assay. For metabolomics and gene expression plant material was collected and immediately frozen until subsequent analysis.

### ^13^C Assay

Plantlets of citrange Carrizo with a single shoot were selected for uniformity of size. The plantlets were transferred to an aerated complemented Hoagland solution for 7 days on hydroponic culture devices for adaptation to hydroponic system. After this period, the Hoagland solution was amended with 1 mM of Hx acid labeled with a ^13^C in the carboxylic end. 48 and 96 h after labeling, the parts of the plant in contact with the solution were washed with 0.1 mM CaSO_4_ in order to avoid any accumulation on the surface of the plant. The roots, stems, and leaves were separated, dried for 48 h at 65°C, crushed in a hammer mill and weighed. The ^13^C analysis was performed using an integrated system for continuous flow isotope ratio mass spectrometry Euro-EA elemental analyzer (EuroVector S.P.A., Milan, Italy) and Isoprime mass spectrometer (GV Instruments, Manchester, UK). The mass of ^13^C was calculated from the fractional abundance (F) and the total C content using a value of 0.0112372 for the absolute isotope ratio, based on the international ^13^C standard Pee Dee Belemite. Values of ^13^C are expressed as δ ^13^C ‰PDB (Stewart and Metherell, 1999).

### LC–ESI Full Scan Mass Spectrometry (Q-TOF Instrument) and Bioinformatics Analysis

The extraction was performed as previously described by Gamir et al. (2012). Aliquots of 20 μL were injected into a UPLC (Waters, Mildford, MA, USA) coupled to a quadrupole-time of flight mass spectrometer (QTOF Premier) through an electrospray ionization source. The LC was developed for 25 min in a common C18 column using a standard variable H_2_O:MeOH gradient. Mass detection was performed using 25 V of cone energy. The drying gas and the nebulizing gas was nitrogen. The desolvation gas flow was set to approximately 600 L/h, and the cone gas flow was set to 60 L/h. A cone voltage of 20 V and a capillary voltage of 3.3 kV were used in the negative ionization mode. The nitrogen desolvation temperature was set at 350°C, and the source temperature was set at 120°C. The instrument was calibrated in the *m*/*z* 50–1000 range with a 1/1 mixture of 0.01 M NaOH/1% HCOOH ten-fold diluted with acetonitrile/water (80/20, v/v).

Raw data obtained from MASSLYNX software were transformed to .CDF format using the DataBridge program provided with the MASSLYNX software. The .CDF data were processed with R for statistical computing using the XCMS package for relative quantification (Smith et al., 2006). For the heat map construction, clustering of metabolite and analysis of results MarVis suite was used^1^ (Kaever et al., 2012). To determine the global behavior of the signals, principal component analyses (PCA) plots were generated using the Multibase 2015 algorithm^2^ In order to identify metabolites, results obtained with QTOF were matched by comparing exact mass with Metlin^3^, KEGG^4^ and MetaCyc databases^5^

### Gene Expression

Gene expression analysis by real-time quantitative PCR (RT-qPCR) was performed. RNA samples were extracted from leaf tissue using the E.Z.N.A. Total RNA Kit II (Omega Bio-Tek; Norcross, GA, USA^6^). Citrus leaf tissue samples for RNA isolation were collected at 96 h post-infection (hpi), and tissues were collected from both treated and non-treated plants. We used the RT-qPCR conditions that were previously described by Flors et al. (2007). Primers were designed using Primer-Blast^7^ for the genes diphosphomevalonate decarboxylase, hydroxymethylglutaryl-CoA reductase, 1-deoxy-D-xylulose-5-phosphate reductoisomerase, hydroperoxide lyase, geranylgeranyl diphosphatase, lipoxygenaseand acetyl-CoA carboxylase 1-like. GAPDH and FBOX were used as internal standard (**Supplementary Table S1**, Primer sequences).

### Volatile Compound Analysis

The extraction was performed as previously described by Beltran et al. (2006) modified for citrus assays. Five gram of entire leaves were collected and introduced directly in a 500 mL Erlenmeyer flask attached to a glass cap with two connection tubes; the inlet was connected to a dry N_2_ gas supply and the outlet to the Tenax trap. Dry nitrogen (99.7%) was used to perform the purge process, and flowed into the flask at 1 L min^-1^. The citrus leaves with 5% (w/w) CaCl_2_ and 50 μL 15 mg mL^-1^ methyl salicylate-D_4_ (surrogate/internal standard) were magnetically stirred (350 rpm) and heated at 35°C for 120 min to enable the volatile analytes to be retained in the Tenax trap (ambient temperature). CaCl_2_ was added to deactivate the enzyme systems. The trap was removed and eluted with 3.5 mL hexane–ether (1:1). The final volume of the extract was adjusted to 1 mL by means of a gentle stream of nitrogen.

### GC–MS

A Varian CP-3800 gas chromatograph coupled with mass spectrometric detector (Saturn 4000, Varian) was used to identify and quantify volatile compounds in the citrus samples. The analytes were separated on a 30 m × 0.25 mm DB-5MS (0.25 μm film thickness) Varian capillary column, with helium at 1 mL min as carrier gas. The temperature program was: 45°C for 5 min, then increased to 96°C at 3°C min^-1^, then increased to 150°C at 6°C min^-1^, and finally increased to 240°C at 30°C min^-1^, with a final isothermal stage of 1.5 min (total chromatographic analysis time 36 min). The gas-chromatograph was directly interfaced with the Varian 4000 mass-spectrometer (ion trap) in the external ionization mode. Quantitation of analytes in the sample extracts was performed using a calibration graph obtained by plotting peak relative area to that of the internal standard methyl salicylate-D_4_ against concentration (ng mL^-1^). m/z ratios used for quantification and confirmation are described by Beltran et al. (2006). The quantification ion used for the internal standard methyl salicylate-D_4_ was 155.

### Statistical Analysis

Statistical analyses were performed using a one-way analysis of variance (ANOVA) in the Statgraphics centurion XVI.I software (Statpoint Technologies, Inc.), and the means were separated using Fisher’s least significant difference (LSD) at 95%. The treatments were 1: non-inoculated untreated plants (control), 2: non-inoculated Hx treated plants (Hx), 3: inoculated untreated plants (Inf), and 4: inoculated Hx treated plants (Hx inf). All of the experiments were repeated three times with six plants per treatment. The figures show the average of three independent experiments.

## Results

### Detection of Hexanoic Acid in Plant after Treatments

**FIGURE 1:**
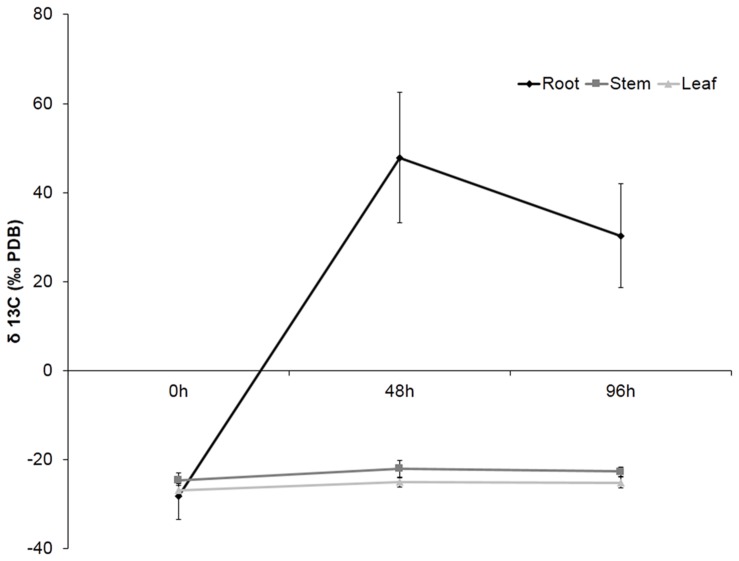
**Recovery of ^13^C in plant tissue.** Roots, stems and leaves were collected at 0, 48, and 96 h after ^13^C pulse labeling. The data show the average of 10 plants per experiment ± SE (*P* < 0.05; least-significant difference test).

In order to determine the location of Hx in the plant or the compounds derived from this molecule after application, plants were treated with Hx labeled with ^13^C on the carboxylic end. The accuracy of the isotope analyzer allowed us to detect the organ with higher concentration of ^13^C. The obtained results showed that, after 48 h, considerable ^13^C accumulation was observed in citrus roots, but the level of labeled carbon was slightly lower at 96 h. However, no ^13^C accumulation was observable in either stems or leaves (**Figure 1**).

### *Alternaria* Disease Severity is Reduced in Treated Plants

**FIGURE 2:**
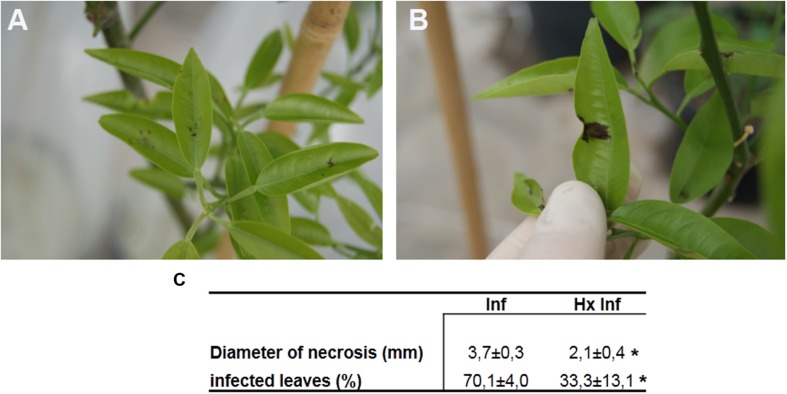
**Effect *Alternaria alternata* on Fortune plants: **(A)** infected plants treated with hexanoic acid and **(B)** untreated infected plants **(C)** disease severity.** The infection diameter measured on 96 h post-inoculation is expressed in mm. Infected leaves expressed as a percentage. Data show the average of three independent experiments obtained with 10 plants per point ± SE. Asterisk in a row represent statistically significant differences (*P* < 0.05; least-significant difference test).

Hexanoic acid treated plants showed significant smaller necrotic lesions in leaves after inoculation with *A. alternata* (**Figures 2A,B**). Ninety six hours after inoculation, untreated plants showed lesions 55% bigger than treated and infected plants. Moreover, the total number of infected leaves was also reduced in treated and infected plants compared with untreated and infected. The number of infected leaves per plant was 47% lower in treated and infected compared with untreated plants (**Figure 2C**).

### Treatment with Hx Acid and Inoculation with *Alternaria alternata* Induce Changes in the Metabolites of Leaves

**FIGURE 3:**
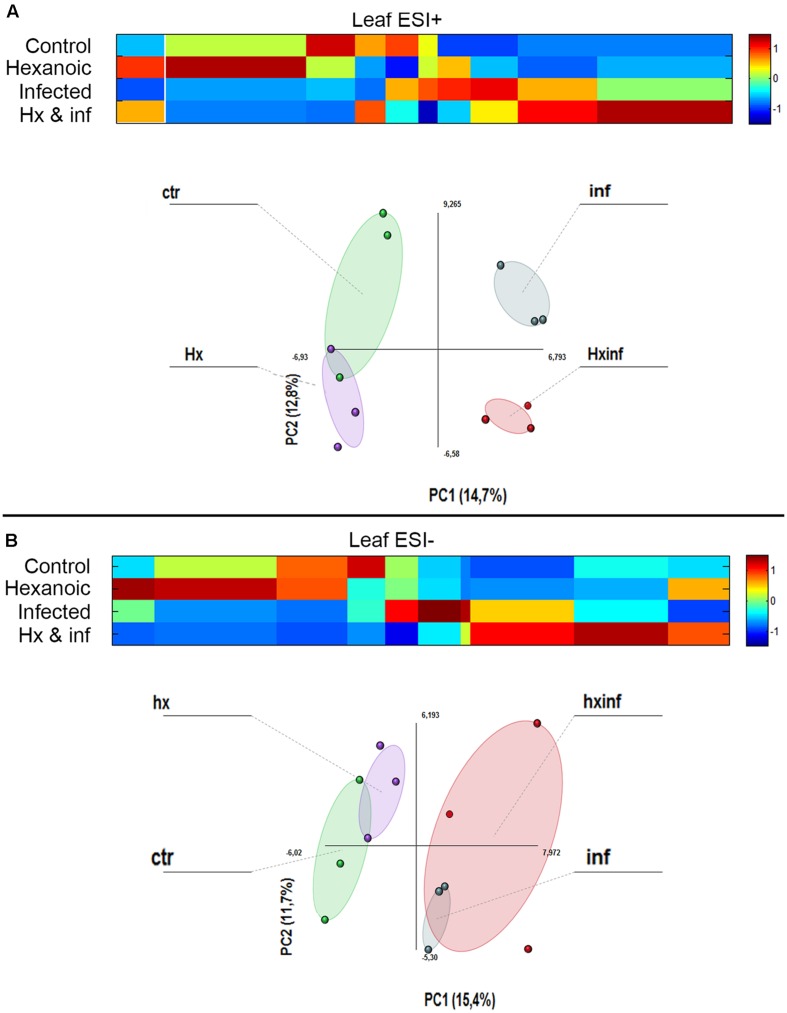
**Principal component analysis (PCA) and cluster plots comparing the signals obtained in ESI + and ESI – for the four treatments: control (Green cicle), Hx (purple circle), infected (blue circle) and Hx and infected (red circle).** The PCA was performed using Multibase package and cluster analyses was performed using Marvis Filter and Cluster packages, following a Kruskal–Wallys test (*P* < 0.05). Cluster analysis show metabolites induced (red color in the scale) or represed (blue color in the scale) for each treatment. **(A)** ESI+ PCA analysis score plot and Cluster plot of main compounds of the four groups 96 h after inoculation. **(B)** ESI- PCA analysis score plot and Cluster plot of main compounds of the four groups 96 h after inoculation.

The metabolomics data obtained by LC-ESI full scan mass spectrometry were processed and submitted to bioinformatics and statistical analyses of the signals. PCA analysis showed strong differences in the behavior of the four treatments, indicating strong changes in the metabolomics profile (**Figure 3**). Results obtained after the Kruskal–Wallys test (*p* < 0.05) indicate that at least 224 compounds were differentially altered in leaves in the positive electrospray ionization mode, whereas 131 compounds were differentially altered in leaves in the negative electrospray ionization mode (**Supplementary Table S2**, List of detected compounds in the LC-ESI analysis).

These compounds were grouped depending on the treatment that produced the alteration. We observe that in leaves, many compounds were altered by Hx application in both the positive (51 compounds) and negative (26 compounds) ionization modes, but only 14 and 4 compounds, respectively, were induced by this treatment application in roots.

When we observed the compounds altered with infection by *A. alternata* in leaves, 17 and 16 compounds were detected in ESI+ and ESI-. The combined effect of treatment and infection on the metabolomics profile was strong. In leaves, 49 and 20 compounds were enhanced only in the Hx and infected plants in the positive and the negative ionization, respectively.

### Global Analysis of Pathways Involved in Hx-IR

MarVis-Pathway interface, allow us to perform a putative annotation of the filtered data obtained in the bioinformatics analysis in the context of plant pathway databases. The analysis of the clusters that represent the compounds altered in each treatment, showed significant matches with several pathways. Those pathways include monoterpenoid, diterpenoid, triterpenoid biosynthesis, terpenoid backbone biosynthesis, limonene and pinene degradation and alpha linoleic acid metabolism. Moreover, the carbon metabolism also seems to be altered (**Table 1**; **Supplementary Figures 1** and **2**).

**Table 1:** Putative identification of pathways altered with the Hx acid treatment (Hx), inoculation with *Alternaria alternata* (inf) and Hx acid treatment and inoculation with *A. alternata.*

Putative pathway	Number of metabolites altered			Ionization
		
	Hx	Inf	Hx Inf	
Terpenoid backbone biosynthesis	0	0	2	ES+
Monoterpenoid biosynthesis	0	0	15	ES+
Diterpenoid biosynthesis	1	0	21	ES+
Limonene and pinene degradation	0	0	11	ES+
Alpha linolenic acid metabolism	3	1	3	ES+
Pentose phosphate pathway	0	0	6	ES+
Carbon metabolism	0	0	5	ES+
Terpenoid backbone biosynthesis	0	0	2	ES-
Diterpenoid biosynthesis	0	0	13	ES-
Alpha linolenic acid metabolism	0	3	4	ES-


*Pathways were identified by exact mass using MarVis suite.*

Despite this identification only gave us a putative list of pathways that can be involved in the Hx-IR, results obtained highlight the implication of terpenoid-derived compounds and linolenic acid derived compounds.

### Individually Putative Identification of Jasmonic and Geranyl-PP Acid Intermediates

The results obtained in the Marvis Pathway analysis indicate that the JA and mevalonic pathways can be altered. In order to confirm this involvement, we performed and in-deep study in order to search the precursors of these pathways between the compounds altered by treatment and/or inoculation.

**FIGURE 4:**
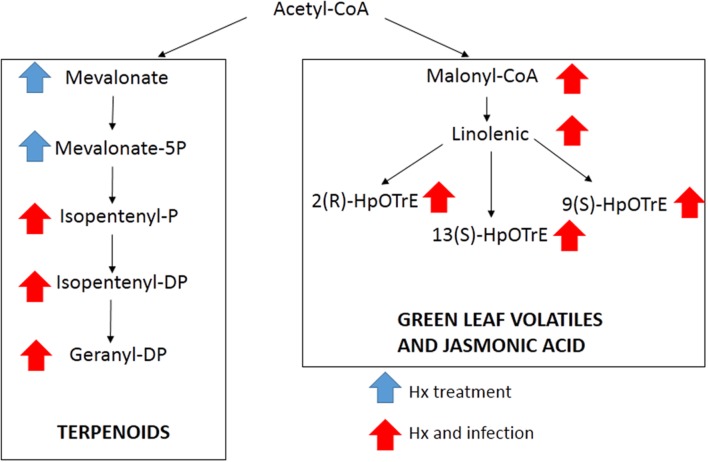
**Compounds putatively identified by exact mass present in mevalonic and linolenic pathways.** Arrows indicate the treatment that showed significant differences.

The comparison of our results with databases, allowed us to identify by exact mass some compounds from the linolenic acid pathway, such as malonyl-CoA, linoleic acid, as well as three compounds with the same m/z, but with a different retention time, that could match the three isomers of HpOTrE (**Figure 4**). The signal obtained for these compounds indicated that this pathway was strongly induced in both the treated and inoculated plants. In the mevalonic acid pathway, the application of Hx in the absence of inoculation enhanced the compounds that matched the m/z of mevalonic acid and mevalonate-5P, while the compounds located downstream were enhanced in the Hx-treated and inoculated plants (**Figure 4**). However, compounds found that could match with the MEP pathway didn’t show significant alteration.

### Gene Expression

**FIGURE 5:**
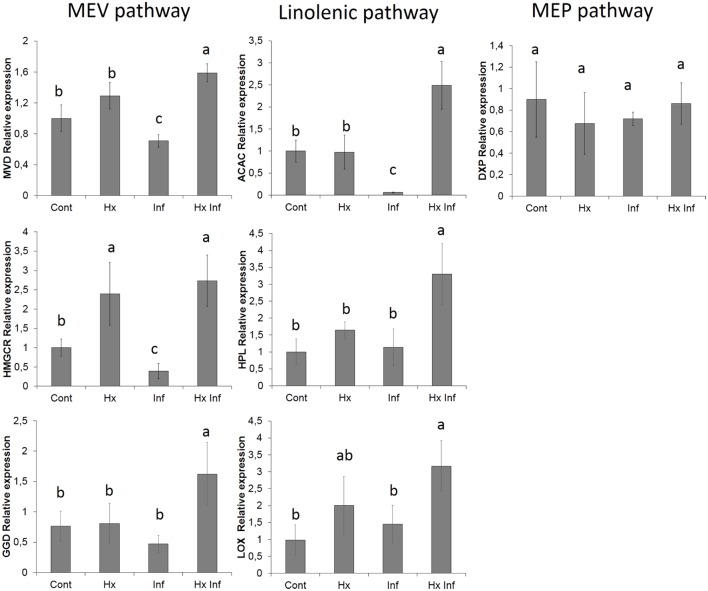
**Effect of different treatments on the relative gene expression.** Marker genes of MEV pahtway (*MVD, HMGCR* and *GGD*), Linolenic pathway (*ACAC, HPL* and *LOX*) and MEP pawthway (*DXP*) Total RNA was isolated from leaves at 96 h post-inoculation and was converted into cDNA and subjected to an RT-qPCR analysis. The results were normalized to the *GAPDH/FBOX* gene expression measured in the same samples. The data show the average of three independent experiments ± SE. Letters indicate statistically significant differences (*P* < 0.05; least-significant difference test).

To corroborate the alteration of the possible implied pathways, the expression of genes present in these pathways was analyzed. For this purpose three genes form the mevalonic acid pathway (diphosphomevalonate decarboxylase, geranylgeranyl diphosphatase and hydroxymethylglutaryl-CoA reductase), three genes from linolenic acid pathway (acetyl-CoA carboxylase 1-like, lipoxygenase and hydroperoxide lyase) and one gene of non-mevalonate pathway as a control (1-deoxy-D-xylulose-5-phosphate reductoisomerase) were chosen. The six genes studied from mevalonic and linolenic pathways showed significant higher expression in treated and infected plants, whereas the marker gene from non-mevalonate pathway didn’t show significant differences between the treatments (**Figure 5**).

### Production of Volatile Compounds is Enhanced by the Treatment with Hx Acid

**FIGURE 6:**
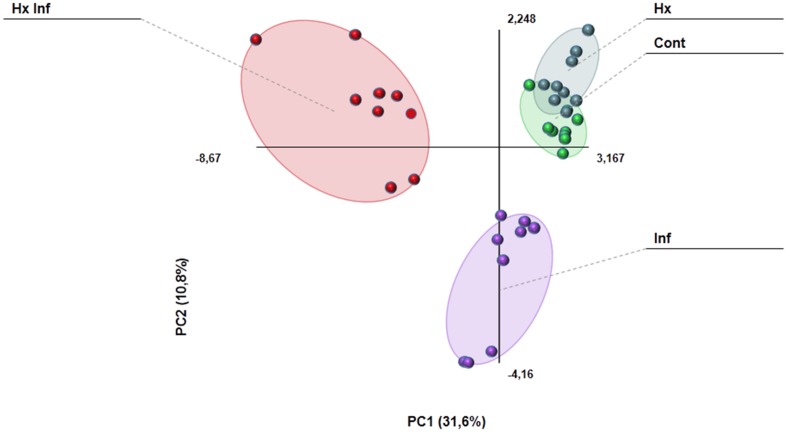
**Principal component analysis comparing the emission of volatile compounds in the different treatments.** The PCA was performed using Multibase package.

The GC-MS analysis allowed us to determine and characterize the most important volatile compounds released by leaves. In our experiment, we detected 27 of the 47 analyzed compounds (**Supplementary Table S3**). Results obtained with the PCA analysis indicates that volatile emission profile is strongly altered in infected, and treated and infected plants, whereas control and hexanoic treated samples are almost overlapped (**Figure 6**). Among the detected compounds, the emission of 17 of them was strongly affected by either treatment, infection or both (**Table 2**). The largest part (13 of these 17 compounds), showed an enhancement of emission in treated and infected plants. On the other hand, only Z-3-hexenol and E-2-heptenal were induced by the treatment in absence of challenge pathogen. Beta ionone was the only compound induced by infection in treated and untreated plants, and ethyl salicylate was the only compound induced in infected plants in absence of treatment. No significant differences between treatments were found for the rest of the detected compounds.

**Table 2:** Volatile compounds detected by GS-MS for the different treatments.

	Control	Hexanoic	Infected	Hexanoic infected
Camphor	N. D.	N. D.	56,3 ± 2,6 ^b^	103,6 ± 16,3^a^
3-Carene	52,6 ± 7,1^b^	40,2 ± 6,8^b^	N. D.	218,4 ± 11,1^a^
Alfa pinene	11172,1 ± 3241,2^b^	4057,2 ± 516,3^b^	7949 ± 1398,6^b^	25355 ± 3978,6^a^
Terpineol	572,8 ± 115,2^c^	1619,3 ± 371,8^bc^	2198,2 ± 292,9^b^	4888,6 ± 599,1^a^
1-Hexanol	104,4 ± 16,5^b^	102,5 ± 14,2^b^	214,4 ± 10,2^b^	784,6 ± 122,9^a^
Gamma terpinene	901,9 ± 126,2^b^	539,8 ± 87,9^c^	386,1 ± 100,4^c^	1291,6 ± 157,1^a^
Z-3-Hexen-1-OL	N. D.	326,3 ± 40,2^a^	N. D.	385 ± 66,8^a^
R-Limonene	5844,4 ± 2278,1^ab^	1917,7 ± 345,9^b^	2978,3 ± 421,8^b^	14197,5 ± 6591,1^a^
2-Carene	153432,6 ± 19994,6^b^	102867,6 ± 35641,6^b^	190481,1 ± 22999,1^b^	666924,6 ± 142808,6^a^
E-2-Heptenal	1128,8 ± 88,8^b^	2681,4 ± 390,8^a^	296,1 ± 33^c^	937,2 ± 166^b^
6-Methyl-5-Hepten-2-OL	306,3 ± 79,4^b^	321,9 ± 131,2^b^	377,3 ± 83,6^b^	951,9 ± 183,1^a^
Beta-Ionone	N. D.	N. D.	105,2 ± 9,8^a^	119,7 ± 10,7^a^
Linalool	49670,4 ± 15698,5^c^	31500,3 ± 5307,8^c^	175073,8 ± 20453^b^	379571,7 ± 72799,9^a^
6-Methyl-5-Hepten-2-one	N. D.	N. D.	151,8 ± 22,8^b^	255,4 ± 36,5^a^
Ethyl salicylate	N. D.	N. D.	163,6 ± 75,2^a^	54,8 ± 41,9^b^
1-Octanol	6064,6 ± 920,5^c^	5977,7 ± 602,3^c^	20544,4 ± 1810,3^b^	43556,5 ± 2545,7^a^
Trans,Trans-2,4-Decadienal	N. D.	N. D.	341 ± 218,2^a^	115,8 ± 40,5^b^


*The data show the average of three independent experiments expressed in pmol/g Fresh weight ± SE. Different letters on the same row indicate significant differences for the compound between treatments. (*P* < 0.05). N.D. means compound not detected.*

## Discussion

The efficacy of Hx as inducer of resistance in plants have been widely studied in the last years. In our lab, we have proved that Hx is capable to induce a primed state of tomato plants against *Alternaria solani* and *Phytophthora citrophthora* (Flors et al., 2003), *arabidopsis* and tomato plants against *Botrytis cinerea*, tomato plants against *Pseudomonas syringae* (Leyva et al., 2008; Vicedo et al., 2009; Kravchuk et al., 2011; Scalschi et al., 2013) and citrus against *Alternaria alternata* and *Xanthomonas citri* (Llorens et al., 2013, 2015b).

Despite this, the knowledge about how Hx operates in plants is very meager. Previous literature was mostly focused on the enhancement of resistance mechanisms. In this way, it is known that against necrotrophic pathogens this compound activates the JA pathway and enhances the callose deposition surrounding the inoculation (Vicedo et al., 2009; Llorens et al., 2013). However, against biotrophic pathogens the application of this acid is also capable of enhance the SA pathway (Scalschi et al., 2013), which implies that the same treatment can induce different molecular changes in plants depending on the pathogen. Moreover, it is also known that plant treatments with Hx can provoke changes in the pathogenesis and survival of the bacteria (Scalschi et al., 2014). For these reasons, in order to study the details of the molecular basis of Hx-IR, we have focused this study on the metabolomics changes induced by the treatment with Hx in presence and absence of the pathogen.

The accuracy of newly developed analysis techniques allowed us to conduct an in-depth study of the alterations caused by this compound in plants during infection. These recent studies demonstrate the potential for using either MS or NMR to obtain detailed information on the relative isotopomer abundance of a wide range of metabolites following labeling with ^13^C -substrates. The ability to take relevant measurements to date, and the application of steady-state labeling strategies to determine metabolic flux, has been largely exploited in plants. Successful studies have applied the labeling method to study the transformation of short chain acids involved in apple flavor, including the Hx (Rowan et al., 1999). Moreover, this technique have also been used to monitorize the changes undergone by a compound in a pathway (Kruger and von Schaewen, 2003; Opitz et al., 2014). The results obtained after treatment with ^13^C-labeled hexanoic provided us the surprising result that the label of this acid was accumulated and remained in roots, and was not detected in either stems or leaves. This result highlights that any protective effect observed by the treatment is independent on the accumulation of the Hx *per se* or any derivative in the leaves, discarding a direct toxic effect on the pathogen. The fact that the labeled carbon stay in the roots may indicate that this molecule is not transported to other parts of the plant, suggesting that can provoke the activation of signal molecules that trigger resistance mechanisms in leaves.

The global analysis of the metabolites altered by the treatment and infection resulted in an elevated number of matches with different terpenoid-related pathways. Which suggest, for the first time, that these molecules can be related with Hx-IR. On the other hand, global analysis of metabolites also indicate that alpha linolenic metabolism can be altered. These results agree with previous ones wich demonstrate that Hx can induce the JA pathway against necrotrophic pathogens and induce the enhancement of compounds such as JA, OPDA or jasmonic-isoleucine and the expression of marker genes such as *AOS, LOX, AOC* or *OPR3* (Vicedo et al., 2009; Camañes et al., 2015).

To confirm the implication of these terpenoids and linolenic derivatives, an individually analysis was performed, searching for all the precursors of these pathways separately. The results provided a putative identification of almost all the intermediates of these pathways, including malonyl-CoA. The involvement of this pathway was corroborated by the enhancement of the expression of lipoxygenase and hydroperoxide lyase genes as well as acetyl-CoA carboxylase gene. This gene is the first step between the acetyl-CoA and the linoleic pathway, which could indicate that the Hx could have an effect near the primary metabolism. On the other hand, isomers of HpOTrE were putatively identified, suggesting that other branches derived from linolenic acid can be involved. Whereas the 13- HpOTrE is the precursor of JA, the isomer 9- HpOTrE is the precursor of green leaf volatiles. The unitary analysis also allowed us to identify the metabolites involved in the mevalonic pathway. The putative identified compounds include compounds as mevalonate and isopentenil, which are precursors of terpenoids, and the enhancement of this pathway was corroborated by the expression of diphosphomevalonate decarboxylase, geranylgeranyl diphosphatase and hydroxymethylglutaryl-CoA reductase. On the other hand, none of the compounds related with the non-mevalonate pathway was significantly altered, and the lack of response in this pathway was confirmed by the analysis of the marker gene 1-deoxy-D-xylulose-5-phosphate reductoisomerase.

A link between volatile compounds and defensive responses, such as enhanced phytoalexins secretion, incorporation of hydroxycinnamic acid esters into the cell wall, enhanced oxidative burst or induction of defense genes, is well-known (Conrath, 2009). It is known that volatiles deriving from linoleic can prime plants for a more robust defense response by increasing total volatiles emission and endogenous JA content after detecting an elicitor (Engelberth et al., 2004). They also seem to increase the sensitivity to methyl jasmonate (MeJA) (Hirao et al., 2012). It has also been reported that treatment with (E)-2-hexenal, (Z)-3-hexenol or (Z)-3-hexenyl acetate induces several defense-related genes, such as chalcone synthase (*CHS*), *AOS*, *HPL* and *LOX2* in *Arabidopsis*, lima beans or citrus (Bate and Rothstein, 1998; Arimura et al., 2001; Gomi et al., 2003), and (Z)-3-hexen-1-ol apparently plays a dual role since it acts both as priming agent in plants and as substrate for toxic compounds against herbivorous insects (Sugimoto et al., 2014). In our experiment, we detected the emission of several volatile compounds derived from the phytooxylipin pathway, suggesting that the HPL branch is strongly induced by the treatment with Hx. Interestingly, the volatiles Z-3-hexenol and E-2-heptenal deriving from this pathway were enhanced with Hx application in the absence of inoculation, suggesting that this pathway is strongly primed by the treatment.

We also observed a second large group of plant volatiles deriving from geranyl diphosphate. These compounds belong to the mevalonate and non-mevalonate pathways, and they are produced mainly from damaged plant leaves (Loughrin et al., 1994; Rose et al., 1996). These volatile compounds also serve to attract pollinators, fruit-dispersing animals and enemies of herbivorous arthropods (Pichersky and Gershenzon, 2002). Some of these compounds are able to activate the expression of a number of defense-related genes, such as *PR2* and *PAL*. All these findings suggest that volatile compounds may act as a signal to facilitate defense responses. Engelberth et al. (2004) also demonstrated that the volatile compounds emitted from herbivore-infested corn plants can prime intact plants against insect herbivore attacks. Moreover, our results agree with the recent studies published by Erb et al. (2015), which observed that linalool is one of the most consistent volatile released by primed plants. However, supporting our results, Yamasaki et al. (2007) and Shishido et al. (2012) demonstrated that, despite the antifungal activity of geranyl diphosphate derivatives, the inoculation of *Alternaria* in citrus, in absence of wounding, is not able to induce strong changes in volatile emission. Despite the implication of terpenes being widely known as players in plant resistance, herein we show for the first time that it might be involved in Hx induced resistance.

Both linolenic and mevalonic pathways start in the primary metabolism, and the results of global pathway analysis showed that carbon metabolism may be implied. In this way, agree with our results, recent studies highlight the implication of the primary metabolism in the priming mediated by β-amino butyric acid (Pastor et al., 2014). Moreover, previous results obtained by Camañes et al. (2015) suggest that Hx-primed and infected plants showed changes in primary metabolism and amino acids, but the metabolites altered were different and pathogen-specific. It has been suggested that Hx as well as the medium chain fatty acid enter the cell rapidly by simple diffusion without implication of specific transporters. Then, Hx is rapidly transformed into an acyl-CoA derivative through the action of a specific acyl-CoA synthetase (Akpa et al., 2010). As a substrate, the presence of Hx in plants has been extensively studied in *Cannabis sativa*, where it is transformed into hexanoyl-CoA (Stout et al., 2012). Taken together, these results could indicate that once inside the plant, Hx could be transformed into a compound that triggers the priming state.

## Conclusion

Results obtained in this study reveal that Hx is absorbed by citrus roots but remains there and it is not further translocated to other parts of the plant. Application of this acid induces resistance against *A. alternata* provoking strong changes in the metabolite profile. With these data, we highlight the alteration of the linolenic and mevalonic pathways, which produce most of the compounds induced by Hx described in this work and in previous ones. Moreover, the possible implication of the carbon metabolism, and the upstream alteration of these two pathways suggest that the priming effect produced by the Hx can take place near the primary metabolism. Which could result in the enhancement of linolenic and mevalonic pathways leading an accumulation of defensive metabolites and the emission of volatile compounds.

## Author Contributions

EL: Design of the work, data acquisition, analysis and interpretation of the results of all the experiments. Drafting, revising and final approval of the manuscript. GC: Data acquisition, analysis and interpretation of the results of Volatile and metabolomics assays. Drafting, revising and final approval of the manuscript. LL: Design of the work, statistical analysis and interpretation of the results. Drafting, revising and final approval of the manuscript. PG-A: Design of the work and interpretation of the results. Drafting, revising and final approval of the manuscript.

## Conflict of Interest Statement

The authors declare that the research was conducted in the absence of any commercial or financial relationships that could be construed as a potential conflict of interest.

## Footnotes

1 http://marvis.gobics.de/

2 http://www.numericaldynamics.com.

3 https://metlin.scripps.edu/index.php

4 www.genome.jp/kegg/

5 MetaCyc.org.

6 http://www.omegabiotek.com

7 http://www.ncbi.nlm.nih.gov/

## References

[B1] Achuo E. A. Audenaert K. Meziane H. Hofte M. (2004). The salicylic acid-dependent defence pathway is effective against different pathogens in tomato and tobacco. *Plant Pathol.* 53 65–72. 10.1111/j.1365-3059.2004.00947.x 12701417

[B2] Akpa M. Point F. Sawadogo S. Radenne A. Mounier C. (2010). Inhibition of Insulin and T3-Induced fatty acid synthase by hexanoate. *Lipids* 45 997–1009. 10.1007/s11745-010-3465-5 20811782

[B3] Aranega Bou P. Leyva M. D. L. O. Finiti I. García Agustín P. González Bosch C. (2014). Priming of plant resistance by natural compounds. Hexanoic acid as a model. *Front. Plant Sci.* 5:488 10.3389/fpls.2014.00488 PMC4181288 25324848

[B4] Arimura G. -I. Ozawa R. Horiuchi J. -I. Nishioka T. Takabayashi J. (2001). Plant–plant interactions mediated by volatiles emitted from plants infested by spider mites. *Biochem. Syst. Ecol.* 29 1049–1061. 10.1016/S0305-1978(01)00049-7

[B5] Bate N. J. Rothstein S. J. (1998). C6-volatiles derived from the lipoxygenase pathway induce a subset of defense-related genes. *Plant J.* 16 561–569. 10.1046/j.1365-313x.1998.00324.x 10036774

[B6] Beltran J. Serrano E. López F. J. Peruga A. Valcarcel M. Rosello S. (2006). Comparison of two quantitative GC–MS methods for analysis of tomato aroma based on purge-and-trap and on solid-phase microextraction. *Anal. Bioanal. Chem.* 385 1255–1264. 10.1007/s00216-006-0410-9 16670892

[B7] Camañes G. Scalschi L. Vicedo B. González-Bosch C. García-Agustín P. (2015). An untargeted global metabolomic analysis reveals the biochemical changes underlying basal resistance and priming in *Solanum lycopersicum*, and identifies 1-methyltryptophan as a metabolite involved in plant responses to *Botrytis cinerea* and *Pseudomonas syringae*. *Plant J.* 84 125–139. 10.1111/tpj.12964 26270176

[B8] Conrath U. (2009). Priming of induced plant defense responses. *Adv. Bot. Res.* 51 361–395. 10.1016/S0065-2296(09)51009-9

[B9] Engelberth J. Alborn H. T. Schmelz E. A. Tumlinson J. H. (2004). Airborne signals prime plants against insect herbivore attack. *Proc. Natl. Acad. Sci. U.S.A.* 101 1781–1785. 10.1073/pnas.0308037100 14749516 PMC341853

[B10] Erb M. Veyrat N. Robert C. A. M. Xu H. Frey M. Ton J. (2015). Indole is an essential herbivore-induced volatile priming signal in maize. *Nat. Commun.* 6:6273 10.1038/ncomms7273. PMC4339915 25683900

[B11] Farmer E. E. (2001). Surface-to-air signals. *Nature* 411 854–856. 10.1038/35081189 11459069

[B12] Flors V. Leyva M. D. L. O. Vicedo B. Finiti I. Real M. D. García-Agustín P. (2007). Absence of the endo-β-1,4-glucanases Cel1 and Cel2 reduces susceptibility to *Botrytis cinerea* in tomato. *Plant J.* 52 1027–1040. 10.1111/j.1365-313X.2007.03299.x 17916112

[B13] Flors V. Miralles C. Cerezo M. Gonzalez-Bosch C. García-Agustín P. (2001). Effect of a novel chemical mixture on senescence processes and plant-fungus interaction in solanaceae plants. *J. Agric. Food Chem.* 49 2569–2575. 10.1021/jf000068y 11368637

[B14] Flors V. Miralles M. C. González-Bosch C. Carda M. García-Agustín P. (2003). Induction of protection against the necrotrophic pathogens *Phytophthora citrophthora* and *Alternaria solani* in *Lycopersicon esculentum* Mill. by a novel synthetic glycoside combined with amines. *Planta* 216 929–938. 10.1007/s00425-002-0945-8 12687360

[B15] Gamir J. Pastor V. Cerezo M. Flors V. (2012). Identification of indole-3-carboxylic acid as mediator of priming against *Plectosphaerella cucumerina*. *Plant Physiol. Biochem.* 61 169–179. 10.1016/j.plaphy.2012.10.004 23116603

[B16] Gomi K. Yamasaki Y. Yamamoto H. Akimitsu K. (2003). Characterization of a hydroperoxide lyase gene and effect of C6-volatiles on expression of genes of the oxylipin metabolism in Citrus. *J. Plant Physiol.* 160 1219–1231. 10.1078/0176-1617-01177 14610891

[B17] Gozzo F. Faoro F. (2013). Systemic Acquired Resistance (50 Years after Discovery): moving from the Lab to the Field. *J. Agric. Food Chem.* 61 12473–12491. 10.1021/jf404156x 24328169

[B18] Heil M. Ton J. (2008). Long-distance signalling in plant defence. *Trends Plant Sci.* 13 264–272. 10.1016/j.tplants.2008.03.005 18487073

[B19] Hirao T. Okazawa A. Harada K. Kobayashi A. Muranaka T. Hirata K. (2012). Green leaf volatiles enhance methyl jasmonate response in *Arabidopsis. J. Biosci. Bioeng*. 114 540–545. 10.1016/j.jbiosc.2012.06.010 22795666

[B20] Jakab G. Ton J. Flors V. Zimmerli L. Metraux J. P. Mauch-Mani B. (2005). Enhancing *Arabidopsis* salt and drought stress tolerance by chemical priming for its abscisic acid responses. *Plant Physiol.* 139 267–274. 10.1104/pp.105.065698 16113213 PMC1203376

[B21] Jung H. W. Tschaplinski T. J. Wang L. Glazebrook J. Greenberg J. T. (2009). Priming in systemic plant immunity. *Science* 324 89–91. 10.1126/science.1170025 19342588

[B22] Kaever A. Landesfeind M. Possienke M. Feussner K. Feussner I. Meinicke P. (2012). MarVis-Filter: ranking, filtering, adduct and isotope correction of mass spectrometry data. *J. Biomed. Biotechnol.* 2012:263910 10.1155/2012/263910 PMC3328170 22550397

[B23] Kravchuk Z. Vicedo B. Flors V. Camañes G. González-Bosch C. García-Agustín P. (2011). Priming for JA-dependent defenses using hexanoic acid is an effective mechanism to protect *Arabidopsis* against *B. cinerea.* *J. Plant Physiol.* 168 359–366. 10.1016/j.jplph.2010.07.028 20950893

[B24] Kruger N. J. von Schaewen A. (2003). The oxidative pentose phosphate pathway: structure and organisation. *Curr. Opin. Plant Biol.* 6 236–246. 10.1016/S1369-5266(03)00039-6 12753973

[B25] Leyva M. O. Vicedo B. Finiti I. Flors V. Del Amo G. Real M. D. (2008). Preventive and post-infection control of *Botrytis cinerea* in tomato plants by hexanoic acid. *Plant Pathol.* 57 1038–1046. 10.1111/j.1365-3059.2008.01891.x

[B26] Loughrin J. H. Manukian A. Heath R. R. Turlings T. C. Tumlinson J. H. (1994). Diurnal cycle of emission of induced volatile terpenoids by herbivore-injured cotton plant. *Proc. Natl. Acad. Sci. U.S.A.* 91 11836–11840. 10.1073/pnas.91.25.11836 11607499 PMC45330

[B27] Llorens E. Fernández-Crespo E. Vicedo B. Lapeña L. García-Agustín P. (2013). Enhancement of the citrus immune system provides effective resistance against Alternaria brown spot disease. *J. Plant Physiol.* 170 146–154. 10.1016/j.jplph.2012.09.018 23260526

[B28] Llorens E. Scalschi L. Fernández-Crespo E. Lapeña L. García-Agustín P. (2015a). Hexanoic acid provides long-lasting protection in ‘Fortune’ mandarin against *Alternaria alternata*. *Physiol. Mol. Plant Pathol.* 91 38–45. 10.1016/j.pmpp.2015.05.005

[B29] Llorens E. Vicedo B. López M. M. Lapeña L. Graham J. H. García-Agustín P. (2015b). Induced resistance in sweet orange against *Xanthomonas citri* subsp. citri by hexanoic acid. *Crop Prot.* 74 77–84. 10.1016/j.cropro.2015.04.008

[B30] Návarová H. Bernsdorff F. Döring A.-C. Zeier J. (2012). Pipecolic acid, an endogenous mediator of defense amplification and priming, is a critical regulator of inducible plant immunity. *Plant Cell* 24 5123–5141. 10.1105/tpc.112.103564 23221596 PMC3556979

[B31] Opitz S. Nes W. D. Gershenzon J. (2014). Both methylerythritol phosphate and mevalonate pathways contribute to biosynthesis of each of the major isoprenoid classes in young cotton seedlings. *Phytochemistry* 98 110–119. 10.1016/j.phytochem.2013.11.010 24359633

[B32] Pastor V. Balmer A. Gamir J. Flors V. Mauch-Mani B. (2014). Preparing to fight back: generation and storage of priming compounds. *Front. Plant Sci.* 5:295 10.3389/fpls.2014.00295 PMC4068018 25009546

[B33] Pastor V. Luna E. Mauch-Mani B. Ton J. Flors V. (2013). Primed plants do not forget. *Environ. Exp. Bot.* 94 46–56. 10.1016/j.envexpbot.2012.02.013

[B34] Pichersky E. Gershenzon J. (2002). The formation and function of plant volatiles: perfumes for pollinator attraction and defense. *Curr. Opin. Plant Biol.* 5 237–243. 10.1016/s1369-5266(02)00251-0 11960742

[B35] Pieterse C. M. J. Van Der Does D. Zamioudis C. Leon-Reyes A. Van Wees S. C. M. (2012). Hormonal modulation of plant immunity. *Annu. Rev. Cell Dev. Biol.* 28 489–521. 10.1146/annurev-cellbio-092910-154055 22559264

[B36] Pieterse C. M. J. Zamioudis C. Berendsen R. L. Weller D. M. Van Wees S. C. M. Bakker P. A. H. M. (2014). Induced systemic resistance by beneficial microbes. *Annu. Rev. Phytopathol.* 52 345–375. 10.1146/annurev-phyto-082712-102340 24906124

[B37] Rose U. Manukian A. Heath R. R. Tumlinson J. H. (1996). Volatile semiochemicals released from undamaged cotton leaves (a systemic response of living plants to caterpillar damage). *Plant Physiol.* 111 487–495. 10.1104/pp.111.2.487 12226304 PMC157859

[B38] Rowan D. D. Allen J. M. Fielder S. Hunt M. B. (1999). Biosynthesis of straight-chain ester volatiles in red delicious and granny smith apples using deuterium-labeled precursors. *J. Agric. Food Chem.* 47 2553–2562. 10.1021/jf9809028 10552526

[B39] Scalschi L. Camañes G. Llorens E. Fernández-Crespo E. López M. M. García-Agustín P. (2014). Resistance inducers modulate *Pseudomonas syringae* pv. *tomato strain DC*3000 response in tomato plants. *PLoS ONE* 9:e106429 10.1371/journal.pone.0106429 PMC4171367 25244125

[B40] Scalschi L. Vicedo B. Camanes G. Fernández-Crespo E. Lapeña L. González-Bosch C. (2013). Hexanoic acid is a resistance inducer that protects tomato plants against *Pseudomonas syringae* by priming the jasmonic acid and salicylic acid pathways. *Mol. Plant Pathol.* 14 342–355. 10.1111/mpp.12010 23279078 PMC6638675

[B41] Shishido H. Miyamoto Y. Ozawa R. Taniguchi S. Takabayashi J. Akimitsu K. (2012). Geraniol synthase whose mRNA is induced by host-selective ACT-toxin in the ACT-toxin-insensitive rough lemon (*Citrus jambhiri*). *J. Plant Physiol.* 169 1401–1407. 10.1016/j.jplph.2012.05.003 22673031

[B42] Smith C. A. Want E. J. O’maille G. Abagyan R. Siuzdak G. (2006). XCMS: processing mass spectrometry data for metabolite profiling using nonlinear peak alignment, matching, and identification. *Anal. Chem.* 78 779–787. 10.1021/ac051437y 16448051

[B43] Stewart D. P. C. Metherell A. K. (1999). Carbon (13C) uptake and allocation in pasture plants following field pulse-labelling. *Plant Soil* 210 61–73. 10.1023/a:1004668910787

[B44] Stout J. M. Boubakir Z. Ambrose S. J. Purves R. W. Page J. E. (2012). The hexanoyl-CoA precursor for cannabinoid biosynthesis is formed by an acyl-activating enzyme in *Cannabis sativa* trichomes. *Plant J.* 71 353–365. 10.1111/j.1365-313X.2012.04949.x 22353623

[B45] Sugimoto K. Matsui K. Iijima Y. Akakabe Y. Muramoto S. Ozawa R. (2014). Intake and transformation to a glycoside of (Z)-3-hexenol from infested neighbors reveals a mode of plant odor reception and defense. *Proc. Natl. Acad. Sci. U.S.A.* 111 7144–7149. 10.1073/pnas.1320660111 24778218 PMC4024874

[B46] Vicedo B. Flors V. Leyva M. D. Finiti I. Kravchuk Z. Real M. D. (2009). Hexanoic acid-induced resistance against *Botrytis cinerea* in tomato plants. *Mol. Plant Microbe Interact.* 22 1455–1465. 10.1094/mpmi-22-11-1455 19810814

[B47] Vicedo B. Leyva M. D. Flors V. Finiti I. Del Amo G. Walters D. (2006). Control of the phytopathogen *Botrytis cinerea* using adipic acid monoethyl ester. *Arch. Microbiol.* 184 316–326. 10.1007/s00203-005-0048-6 16261314

[B48] Winter T. R. Borkowski L. Zeier J. Rostás M. (2012). Heavy metal stress can prime for herbivore-induced plant volatile emission. *Plant Cell Environ.* 35 1287–1298. 10.1111/j.1365-3040.2012.02489.x 22321129

[B49] Yamasaki Y. Kunoh H. Yamamoto H. Akimitsu K. (2007). Biological roles of monoterpene volatiles derived from rough lemon (*Citrus jambhiri* Lush) in citrus defense. *J. Gen. Plant Pathol.* 73 168–179. 10.1007/s10327-007-0013-0

[B50] Yu C. Zeng L. Z. Sheng K. Chen F. X. Zhou T. Zheng X. D. (2014). gamma-Aminobutyric acid induces resistance against *Penicillium expansum* by priming of defence responses in pear fruit. *Food Chem.* 159 29–37. 10.1016/j.foodchem.2014.03.011 24767023

