# Supplementary material for: Priming by Hexanoic Acid Induce Activation of Mevalonic and Linolenic Pathways and Promotes the Emission of Plant Volatiles
Source: Front Plant Sci. 2016 Apr 12;7:495. doi: 10.3389/fpls.2016.00495 (PMC4828442; doi:10.3389/fpls.2016.00495)
Supplement: Supplementary file 1 [file Table_1.DOCX]

Supplementary Material

Priming by Hexanoic acid induce activation of mevalonic and linolenic pathways and promotes the emission of plant volatiles.

Eugenio Llorens*, Gemma Camañes, Leonor Lapeña, Pilar García-Agustín

*** Correspondence:** Dr. Eugenio Llorens: ellorens@uji.es

# Supplementary Table 1: primers used in this work

|  |  | **Sequence (5'->3')** |
| --- | --- | --- |
| **diphosphomevalonate decarboxylase** | **For** | GTGAGTTCACGGCAGAAGGA |
|  | **Rev** | AACAGCATTAGGCCCAGCAT |
| **hydroxymethylglutaryl-CoA reductase** | **For** | TGCGAGGCAACAATCAAGGA |
|  | **Rev** | ACTTAATGCCCAATTACCTCTATGG |
| **acetyl-CoA carboxylase 1-like** | **For** | TTGAAAAGGCCCACCACCTT |
|  | **Rev** | AAGAGAACCAGCAGCTCACG |
| **1-deoxy-D-xylulose-5-phosphate reductoisomerase** | **For** | TCCAAAGACATGGGAAGGTC |
|  | **Rev** | TCGGGCTTTTCTTCAACATT |
| **Hydroperoxide lyase** | **For** | GTGAGTTGCTTTGCGGGTAT |
|  | **Rev** | CGATCAAACAAGCAACGAGA |
| **Geranylgeranyl diphosphate** | **For** | TCGGAGGAGGAAATGAAGAA |
|  | **Rev** | TCGATCCCCACTAACTTTGG |
| **Lipoxygenase** | **For** | AATAGCAACCAATCGGCAAC |
|  | **Rev** | TCCCCGGCTAATTAGGTCTT |
| **GAPDH** | **For** | GGAAGGTCAAGATCGGAATCAA |
|  | **Rev** | CGTCCCTCTGCAAGATGACTCT |
| **FBOX** | **For** | TTGGAAACTCTTTCGCCACT |
|  | **Rev** | CAGCAACAAAATACCCGTCT |
