# Supplementary material for: Priming by Hexanoic Acid Induce Activation of Mevalonic and Linolenic Pathways and Promotes the Emission of Plant Volatiles
Source: Front Plant Sci. 2016 Apr 12;7:495. doi: 10.3389/fpls.2016.00495 (PMC4828442; doi:10.3389/fpls.2016.00495)
Supplement: Supplementary file 2 [file Table_2.DOCX]

Supplementary Material

Priming by Hexanoic acid induce activation of mevalonic and linolenic pathways and promotes the emission of plant volatiles.

Eugenio Llorens*, Gemma Camañes, Leonor Lapeña, Pilar García-Agustín

*** Correspondence:** Dr. Eugenio Llorens: ellorens@uji.es

# Supplementary Table 2: List of detected compounds in the LC-ESI analysis in (A) positive ionization and (B) Negative ionization for the treatments control (Cont), infected (Inf) treated with hexanoic acid (Hx) and treated with hexanoic and infected (Hxinf). Id: putative identification number, rtmed: retention time, mzmed: ratio mass charge. Values of intensity have been transformed and are expressed in log-scale. Red highlightig indicate higher intensities and blue highlightig indicate lower intensities.

| **A** |  |  |  |  |  |  |
| --- | --- | --- | --- | --- | --- | --- |
| **id** | **rtmed** | **mzmed (corrected)** | **Cont** | **Inf** | **hx** | **Hx inf** |
| 1 | 776.954895 | 832.2479248 | 0,711817324 | 1,000328779 | -0,972125292 | -0,740020871 |
| 2 | 1022.039917 | 621.4073486 | 0,952962101 | 0,774274111 | -0,845041037 | -0,882195234 |
| 3 | 1072.221436 | 586.4510498 | 1,060896277 | 0,646140039 | -0,850735843 | -0,856300533 |
| 4 | 539.5142212 | 792.5234375 | 0,854434907 | 0,836693466 | -0,581057429 | -1,110070825 |
| 5 | 1222.25769 | 785.5977783 | 1,235606551 | 0,363112837 | -0,621211708 | -0,97750777 |
| 6 | 374.3243713 | 436.2295532 | 0,60582 | 0,940514982 | -0,247411981 | -1,298923016 |
| 7 | 663.2301025 | 584.390564 | 0,633038759 | 0,884800792 | -0,182521045 | -1,335318565 |
| 8 | 1258.748657 | 236.3618774 | 0,181455433 | 1,07405591 | 0,088248305 | -1,343759656 |
| 9 | 739.8258667 | 717.2767334 | 1,147137761 | 0,332638204 | -0,250726342 | -1,229049683 |
| 10 | 1191.674438 | 497.3534851 | -0,4858329 | 1,295516133 | 0,2107113 | -1,020394564 |
| 11 | 1095.543579 | 698.3614502 | 1,070454836 | 0,273499578 | -0,010079199 | -1,333875179 |
| 12 | 671.3721924 | 661.2280273 | 1,25378859 | 0,060990989 | -0,128252968 | -1,186526656 |
| 13 | 893.0895386 | 303.2935486 | -0,031946227 | 0,975851655 | 0,422813773 | -1,366719246 |
| 14 | 1185.230347 | 795.5518188 | 0,678798735 | 0,56134373 | 0,232908502 | -1,473050952 |
| 15 | 1216.134521 | 580.8935547 | 1,078415036 | 0,186953947 | 0,074948795 | -1,340317845 |
| 16 | 669.3577271 | 600.2470703 | 1,137596726 | 0,09340097 | 0,069874153 | -1,300871849 |
| 17 | 987.2850952 | 621.2722778 | -0,300800651 | 0,945325553 | 0,628571987 | -1,273096919 |
| 18 | 1137.60022 | 627.5073853 | 0,026141007 | 0,756864369 | 0,638041914 | -1,42104733 |
| 19 | 532.0444336 | 566.4244995 | 0,362290323 | 0,507299423 | 0,621921718 | -1,491511464 |
| 20 | 770.3103027 | 379.6522827 | -0,422779918 | 0,304597139 | 1,225602031 | -1,107419252 |
| 21 | 752.7679443 | 883.4476318 | 0,131209314 | 0,021805637 | 1,142221808 | -1,295236707 |
| 22 | 766.5354004 | 206.1301727 | -0,580106556 | 0,05769214 | 1,384525895 | -0,862111449 |
| 23 | 644.4888306 | 163.0313721 | 0,946072996 | -0,068831719 | 0,487508923 | -1,364750147 |
| 24 | 693.1162109 | 496.1773987 | 0,356465668 | -0,142465174 | 1,082482338 | -1,296482921 |
| 25 | 1254.297485 | 550.3695679 | 0,815666378 | -0,164184168 | 0,697859705 | -1,349341989 |
| 26 | 585.4675903 | 170.091217 | 0,194172323 | -0,267892003 | 1,238486648 | -1,164766908 |
| 27 | 547.5829468 | 250.1172028 | 0,458402008 | -0,290028274 | 1,075894237 | -1,244267941 |
| 28 | 741.2906494 | 266.1531067 | 0,599019826 | -0,331277967 | 0,983175874 | -1,250917792 |
| 29 | 691.4631958 | 309.1630554 | 0,254536688 | -0,35259077 | 1,23352766 | -1,135473609 |
| 30 | 735.0462646 | 194.1302948 | 0,49232313 | -0,359655201 | 1,078101635 | -1,210769534 |
| 31 | 690.9971924 | 268.167572 | 0,378868461 | -0,377351284 | 1,164158463 | -1,16567564 |
| 32 | 713.6140137 | 299.1785889 | 0,374697357 | -0,430661529 | 1,183959246 | -1,127995133 |
| 33 | 735.0006104 | 234.1222992 | 0,48206839 | -0,465337038 | 1,120999575 | -1,137730837 |
| 34 | 734.3005371 | 622.2362061 | 0,513150096 | -0,464745373 | 1,098187327 | -1,146592021 |
| 35 | 1007.642578 | 491.11026 | 1,054144263 | -0,419505984 | 0,551994383 | -1,186632633 |
| 36 | 589.9483643 | 736.3063354 | 0,297332108 | -0,503338158 | 1,251268029 | -1,045261979 |
| 37 | 674.4437256 | 527.2235718 | 1,085000396 | -0,435955524 | 0,518711567 | -1,167756438 |
| 38 | 734.7792969 | 462.2374268 | 0,022329545 | -0,524680316 | 1,390919566 | -0,888568819 |
| 39 | 715.4325562 | 670.2971191 | 0,375099003 | -0,550189078 | 1,214768291 | -1,039678216 |
| 40 | 544.2342529 | 476.2967529 | 0,520344257 | -0,572357118 | 1,121519208 | -1,069506407 |
| 41 | 583.9293213 | 540.8286133 | 0,478463292 | -0,649412632 | 1,165920019 | -0,994970679 |
| 42 | 712.4552612 | 298.175354 | 0,491354942 | -0,686325491 | 1,162501931 | -0,967531383 |
| 43 | 581.6286621 | 1073.668701 | 0,558106899 | -0,683369637 | 1,11469543 | -0,989432573 |
| 44 | 456.4126892 | 268.1288147 | 0,596857309 | -0,682311535 | 1,085454583 | -1,000000358 |
| 45 | 764.1450195 | 726.2158203 | 0,708049536 | -0,650120616 | 0,989448786 | -1,047377706 |
| 46 | 606.7994385 | 285.1657104 | 0,47893393 | -0,743198097 | 1,176967263 | -0,912703097 |
| 47 | 544.4101563 | 465.7977295 | 0,643001556 | -0,697117627 | 1,051536322 | -0,997420311 |
| 48 | 604.760376 | 284.1604919 | 0,538419068 | -0,762625694 | 1,137620926 | -0,9134143 |
| 49 | 687.2647095 | 192.1147766 | 0,720659733 | -0,698928416 | 0,987110436 | -1,008841753 |
| 50 | 1247.135864 | 726.5856323 | 0,633735895 | -0,735245585 | 1,063829541 | -0,962319851 |
| 51 | 544.4101563 | 465.2946472 | 0,528176427 | -0,786709964 | 1,146296144 | -0,887762547 |
| 52 | 545.4678345 | 211.1292114 | 0,406949639 | -0,810655415 | 1,225508213 | -0,821802497 |
| 53 | 545.6781006 | 210.1246338 | 0,545723319 | -0,805332065 | 1,134774089 | -0,875165284 |
| 54 | 546.5342407 | 475.7938538 | 0,637438536 | -0,799337089 | 1,06624794 | -0,904349327 |
| 55 | 582.1864624 | 227.1283112 | 0,509949327 | -0,841015399 | 1,159978271 | -0,828912139 |
| 56 | 583.102356 | 547.8174438 | 0,382709414 | -0,869667232 | 1,238126636 | -0,751168907 |
| 57 | 785.442688 | 1143.380981 | 0,772052229 | -0,831828475 | 0,95460701 | -0,894830763 |
| 58 | 547.1970825 | 251.1221619 | 0,515363574 | -0,912126422 | 1,153293848 | -0,756530941 |
| 59 | 736.1641235 | 195.1338348 | 0,197050497 | -0,924077511 | 1,323453069 | -0,59642601 |
| 60 | 546.0010376 | 494.2236633 | 0,513361216 | -0,916531384 | 1,154254317 | -0,751084149 |
| 61 | 1264.138794 | 408.1221924 | 0,548765183 | -0,911826968 | 1,130614996 | -0,76755327 |
| 62 | 686.6656494 | 232.1077728 | 0,775893211 | -0,85062182 | 0,951596201 | -0,876867652 |
| 63 | 582.677063 | 530.3387451 | 0,454238087 | -0,937350929 | 1,189802647 | -0,706689835 |
| 64 | 458.8272705 | 340.1490479 | 0,215341613 | -0,948626578 | 1,311333537 | -0,578048527 |
| 65 | 586.2268066 | 266.1131592 | 0,56765008 | -0,94759953 | 1,114000678 | -0,734051168 |
| 66 | 585.2576294 | 267.1172485 | 0,485285848 | -0,964337587 | 1,166835308 | -0,687783539 |
| 67 | 547.4733276 | 483.2983093 | 0,40103206 | -0,971332371 | 1,216027498 | -0,645727277 |
| 68 | 799.366272 | 396.4844055 | 0,104509875 | -0,962335289 | 1,349525571 | -0,491700172 |
| 69 | 785.0014038 | 1142.371094 | 0,890778005 | -0,89059478 | 0,840549648 | -0,840732873 |
| 70 | 589.0922241 | 737.3139038 | 0,371521711 | -1,023357511 | 1,22097826 | -0,569142461 |
| 71 | 735.4030151 | 148.1244507 | 0,380181402 | -1,039257765 | 1,212208748 | -0,553132415 |
| 72 | 544.0654297 | 944.5999146 | 0,544936836 | -1,03883028 | 1,113523364 | -0,61962992 |
| 73 | 780.1845703 | 1160.381836 | 0,915258586 | -1,014340401 | 0,80070889 | -0,701627076 |
| 74 | 704.0582886 | 459.6555176 | 0,512728334 | -1,138028145 | 1,102112293 | -0,476812542 |
| 75 | 500.7063293 | 281.0574951 | 1,201291561 | -0,812458575 | 0,446369797 | -0,835202813 |
| 76 | 623.0542603 | 525.3847656 | 1,231885672 | -1,077586532 | 0,316144854 | -0,470443934 |
| 77 | 1134.827393 | 785.4973145 | 0,65072006 | -1,369578958 | 0,829768598 | -0,110909723 |
| 78 | 1277.393433 | 465.1951294 | 0,76131928 | -1,37507844 | 0,71991843 | -0,106159337 |
| 79 | 477.8450317 | 317.6869812 | 0,166895688 | -1,352507472 | 1,061864376 | 0,123747468 |
| 80 | 362.7629395 | 322.0802917 | 0,965612352 | -1,341910124 | 0,501254261 | -0,124956392 |
| 81 | 592.8045044 | 399.8031006 | 0,014408816 | -1,317798495 | 1,106550455 | 0,196839228 |
| 82 | 1238.666016 | 311.3235168 | 1,043089032 | -1,332795262 | 0,361252308 | -0,071546145 |
| 83 | 988.210022 | 466.8738403 | -0,06734135 | -1,314571977 | 1,086256146 | 0,295657158 |
| 84 | 1199.856689 | 1049.544434 | 1,345018029 | -1,073949456 | -0,155781761 | -0,115286738 |
| 85 | 767.5357666 | 452.8340759 | 0,353747249 | -1,482907891 | 0,703045368 | 0,426115304 |
| 86 | 685.4680786 | 158.1488342 | 0,195030704 | -1,454970837 | 0,790123463 | 0,469816685 |
| 87 | 974.2402954 | 244.0717621 | 1,466127157 | -0,694876254 | -0,572980762 | -0,198270232 |
| 88 | 793.6413574 | 463.9944458 | 1,118007541 | -1,100796819 | -0,527333319 | 0,510122597 |
| 89 | 611.3110352 | 943.6413574 | 0,866384804 | -1,153943539 | -0,518204987 | 0,805763722 |
| 90 | 1219.089355 | 750.9100342 | 0,786516368 | -1,268631101 | -0,331546336 | 0,813661039 |
| 91 | 1277.254272 | 354.2698364 | 0,877041817 | -1,389443636 | -0,034458715 | 0,546860576 |
| 92 | 789.8388062 | 654.2641602 | 0,32248497 | -0,864131033 | -0,729824424 | 1,271470547 |
| 93 | 874.7265625 | 879.9736328 | 0,115787908 | -0,845922887 | -0,636046648 | 1,366181612 |
| 94 | 570.5769653 | 166.099472 | 0,332412541 | -1,259758115 | -0,196829572 | 1,124175072 |
| 95 | 1202.559082 | 1079.827881 | 0,314576745 | -1,326912999 | -0,054158077 | 1,066494346 |
| 96 | 536.0611572 | 768.4815063 | 0,390311182 | -1,431350708 | 0,162043423 | 0,878996134 |
| 97 | 1007.915649 | 465.2678833 | 0,039127573 | -1,417064428 | 0,545715988 | 0,832220852 |
| 98 | 1013.190308 | 101.9825287 | -0,147951454 | -1,287444353 | 0,336635739 | 1,098760128 |
| 99 | 1213.184448 | 635.3546143 | -0,413430274 | -1,188782215 | 0,543880701 | 1,058331728 |
| 100 | 478.0894775 | 568.4333496 | -0,64266777 | -1,028883219 | 0,579517424 | 1,092033505 |
| 101 | 875.1083374 | 157.0191803 | -0,56678611 | -1,110071898 | 0,980849683 | 0,696008384 |
| 102 | 715.9318848 | 746.0327148 | -0,689641118 | -1,025291681 | 0,820894539 | 0,8940382 |
| 103 | 801.7335205 | 210.0856171 | -0,90071094 | -0,640378416 | 0,226873308 | 1,314216018 |
| 104 | 1268.359009 | 892.6591797 | -0,945451677 | -0,766501784 | 1,019077539 | 0,692875862 |
| 105 | 787.000061 | 268.2053528 | -0,634239733 | -0,886999548 | 1,332409501 | 0,188829765 |
| 106 | 303.693573 | 361.2371521 | -0,770593047 | -0,829067648 | 1,26853931 | 0,331121355 |
| 107 | 1112.038452 | 502.3472595 | -1,074289083 | -0,623331606 | 0,939371109 | 0,758249641 |
| 108 | 1196.682861 | 778.5359497 | -1,304699898 | -0,259086728 | 0,718972683 | 0,844813883 |
| 109 | 1291.234619 | 874.2697754 | -1,247447491 | -0,290744841 | 1,066017389 | 0,472174943 |
| 110 | 524.6782837 | 587.4031372 | -0,771153092 | 0,085282989 | 1,38284874 | -0,696978569 |
| 111 | 664.7471313 | 533.2545776 | -0,733978391 | 0,674975336 | 1,030486822 | -0,971483827 |
| 112 | 1220.285889 | 244.0711365 | -1,02764082 | 0,579780161 | 1,092123747 | -0,644263148 |
| 113 | 1106.386841 | 386.2909851 | -0,851206422 | 0,997083426 | 0,724128723 | -0,870005727 |
| 114 | 647.3856201 | 180.1143799 | -1,339473486 | 0,219028533 | 1,075071454 | 0,045373496 |
| 115 | 1106.752563 | 444.293457 | -1,359144568 | 0,87041539 | 0,615629196 | -0,126900002 |
| 116 | 599.7572632 | 409.2058411 | -1,177566409 | 1,134535909 | 0,424776971 | -0,381746441 |
| 117 | 781.7524414 | 831.6572266 | -1,428167939 | 0,669491768 | 0,714242935 | 0,04443321 |
| 118 | 831.2698975 | 770.2369385 | -1,39720118 | 0,868846953 | 0,541083992 | -0,012729837 |
| 119 | 493.7577209 | 590.1783447 | -1,350447178 | 0,979476571 | 0,457614303 | -0,086643629 |
| 120 | 1077.374756 | 170.0934753 | -1,481259823 | 0,270929873 | 0,602255046 | 0,608074904 |
| 121 | 1045.753662 | 736.4742432 | -1,41805172 | 0,931131125 | 0,285757899 | 0,20116277 |
| 122 | 958.0928955 | 362.1012573 | -1,467309356 | 0,780883908 | 0,314634472 | 0,371791035 |
| 123 | 1245.237793 | 572.4229126 | -0,608504355 | 1,486912847 | -0,56768018 | -0,310728282 |
| 124 | 610.8079834 | 907.6906738 | -1,152224183 | 1,215636492 | -0,342025876 | 0,278613597 |
| 125 | 349.586853 | 204.0602875 | -1,415699124 | 0,814061523 | 0,025032863 | 0,576604724 |
| 126 | 582.1376953 | 417.0289001 | -1,363866329 | 0,857313216 | -0,118600354 | 0,625153363 |
| 127 | 667.1481323 | 490.6939697 | -1,381171107 | 0,804403245 | -0,085093684 | 0,661861599 |
| 128 | 586.0947266 | 220.108078 | -1,254868388 | 1,003742337 | -0,313910753 | 0,565036774 |
| 129 | 347.3814697 | 278.0230103 | -1,063589334 | 1,171627879 | -0,549109995 | 0,44107151 |
| 130 | 318.3236084 | 598.3857422 | -1,16437912 | 1,022256613 | -0,471669376 | 0,613791883 |
| 131 | 1121.443604 | 1005.746948 | -1,440510154 | 0,360465646 | 0,214640021 | 0,865404427 |
| 132 | 509.8022156 | 582.3187256 | -1,439187407 | 0,365259856 | 0,206309423 | 0,867618144 |
| 133 | 801.3970947 | 150.1033783 | -1,362378359 | 0,651944935 | -0,127761141 | 0,838194549 |
| 134 | 1253.672241 | 663.9279785 | -1,214566112 | 0,914499044 | -0,4171969 | 0,717263937 |
| 135 | 788.6581421 | 307.1687012 | -1,056889296 | 1,037876368 | -0,625165463 | 0,644178391 |
| 136 | 469.7195435 | 176.0800934 | -1,069731236 | 1,011623979 | -0,615385592 | 0,673492789 |
| 137 | 747.8392944 | 400.1611328 | -1,390889883 | 0,078199513 | 0,341915369 | 0,970775008 |
| 138 | 297.7286682 | 268.0563049 | -1,376039267 | 0,41607824 | -0,006142952 | 0,966104031 |
| 139 | 441.3637085 | 295.1217346 | -0,913024426 | 1,103932261 | -0,777178586 | 0,58627075 |
| 140 | 441.5456543 | 335.1150818 | -0,973150909 | 1,04703033 | -0,727582812 | 0,653703392 |
| 141 | 818.0899658 | 699.3294678 | -1,26435554 | 0,584854364 | -0,303867936 | 0,983369112 |
| 142 | 451.3184814 | 238.1215668 | -1,240444779 | 0,620297968 | -0,354846239 | 0,97499305 |
| 143 | 369.0705566 | 239.0943451 | -1,038541317 | 0,829877913 | -0,673795641 | 0,882459045 |
| 144 | 374.345459 | 385.0812988 | -1,165849686 | 0,620683134 | -0,471373528 | 1,01654017 |
| 145 | 496.5933228 | 468.2288513 | -1,230407 | 0,351385325 | -0,259161979 | 1,138183713 |
| 146 | 337.5527039 | 186.0286713 | -1,089375973 | 0,132655203 | -0,339565486 | 1,296286225 |
| 147 | 374.3013611 | 385.0395203 | -0,996289909 | -0,003071256 | -0,368661404 | 1,368022561 |
| 148 | 814.1861572 | 620.4210815 | -1,115172505 | 0,267361134 | -0,390193909 | 1,23800528 |
| 149 | 814.569458 | 712.3539429 | -1,100958943 | 0,247329176 | -0,398526281 | 1,252156019 |
| 150 | 336.9546814 | 384.0727234 | -1,110769272 | 0,391804755 | -0,463386416 | 1,182350874 |
| 151 | 813.7371826 | 270.2196045 | -1,106860876 | 0,410065264 | -0,477418065 | 1,174213648 |
| 152 | 814.0178223 | 311.216156 | -1,089028716 | 0,404607266 | -0,499244273 | 1,183665752 |
| 153 | 814.1861572 | 271.2225037 | -1,08027339 | 0,395726711 | -0,506875575 | 1,191422224 |
| 154 | 488.5449219 | 424.2140808 | -1,074404359 | 0,40782392 | -0,520281851 | 1,18686223 |
| 155 | 337.7724609 | 201.9939575 | -0,969953775 | 0,197008759 | -0,541335404 | 1,31428051 |
| 156 | 423.6819458 | 134.1091003 | -1,017960429 | 0,356282264 | -0,568580389 | 1,230258465 |
| 157 | 506.0922546 | 397.1906128 | -1,047971129 | 0,440439642 | -0,568937659 | 1,176469088 |
| 158 | 814.1861572 | 234.1961212 | -1,120209694 | 0,602027476 | -0,530983806 | 1,049165964 |
| 159 | 814.0839844 | 312.2174072 | -1,077949524 | 0,516341865 | -0,559944093 | 1,121551752 |
| 160 | 372.0240173 | 386.0857239 | -0,946171582 | 0,253049523 | -0,602258325 | 1,295380354 |
| 161 | 337.7147827 | 147.0405579 | -0,81716609 | -0,020953853 | -0,576072633 | 1,414192677 |
| 162 | 813.526123 | 310.2121582 | -1,005543232 | 0,400376767 | -0,604463577 | 1,209630132 |
| 163 | 1195.312012 | 308.2139587 | -1,096363068 | 0,610777974 | -0,565613926 | 1,051198959 |
| 164 | 856.8299561 | 838.3363037 | -1,069628239 | 0,606419742 | -0,599319756 | 1,062528253 |
| 165 | 499.8942871 | 268.2209778 | -1,041784167 | 0,55654937 | -0,619708479 | 1,104943395 |
| 166 | 813.3803711 | 728.3392334 | -1,073846102 | 0,639107823 | -0,60235554 | 1,037093759 |
| 167 | 818.2510986 | 698.3265991 | -1,053422928 | 0,596347749 | -0,617101491 | 1,07417655 |
| 168 | 376.2781677 | 328.0904846 | -1,033930063 | 0,56813103 | -0,633048296 | 1,098847389 |
| 169 | 497.9224548 | 254.0835114 | -0,910330772 | 0,295078874 | -0,665759623 | 1,281011462 |
| 170 | 372.8131714 | 601.1582642 | -0,876684964 | 0,262441665 | -0,686331987 | 1,300575376 |
| 171 | 337.1168823 | 352.0560608 | -0,699407518 | -0,029241247 | -0,695040882 | 1,423689604 |
| 172 | 500.3946228 | 216.1886749 | -0,993338287 | 0,636104643 | -0,700258017 | 1,05749166 |
| 173 | 813.0385742 | 744.3103027 | -0,909932673 | 0,450825751 | -0,735689819 | 1,194796681 |
| 174 | 494.3170776 | 375.1645508 | -0,859048426 | 0,366017968 | -0,755115271 | 1,2481457 |
| 175 | 506.1531067 | 414.1590881 | -0,911419332 | 0,531771481 | -0,762664318 | 1,142312169 |
| 176 | 813.3597412 | 253.2111053 | -0,806487262 | 0,35529092 | -0,804343045 | 1,255539298 |
| 177 | 813.9215088 | 344.1491394 | -0,896628678 | 0,599620879 | -0,798175812 | 1,095183611 |
| 178 | 812.0836792 | 614.4060059 | -0,742884338 | 0,281713516 | -0,832998395 | 1,294169188 |
| 179 | 354.7595825 | 254.0844421 | -0,883790076 | 0,613648772 | -0,815156162 | 1,085297465 |
| 180 | 501.1872559 | 547.3455811 | -0,80988574 | 0,455346674 | -0,841036797 | 1,195575833 |
| 181 | 419.0693665 | 190.0966339 | -0,787191629 | 0,432465524 | -0,854719222 | 1,209445357 |
| 182 | 355.5016479 | 246.0515289 | -0,814401865 | 0,494461477 | -0,850246728 | 1,170187116 |
| 183 | 501.9030762 | 548.3414917 | -0,804280102 | 0,474334985 | -0,853287756 | 1,183232784 |
| 184 | 375.8653564 | 215.0563965 | -0,737517715 | 0,360681891 | -0,87341392 | 1,250249743 |
| 185 | 624.6365356 | 257.1061401 | -0,92506212 | 0,835006893 | -0,804374695 | 0,894429922 |
| 186 | 376.4252014 | 210.0464478 | -0,834762096 | 0,726739168 | -0,886517048 | 0,994540036 |
| 187 | 355.2461548 | 145.0521393 | -0,741315424 | 0,52003634 | -0,927515388 | 1,148794532 |
| 188 | 489.6311951 | 171.0917969 | -0,808134437 | 0,67864877 | -0,904269874 | 1,033755541 |
| 189 | 1135.484741 | 395.7903748 | -0,375950635 | -0,267902762 | -0,813770354 | 1,45762372 |
| 190 | 385.1625061 | 180.0233154 | -0,497710705 | 0,011749509 | -0,904640198 | 1,390601397 |
| 191 | 892.2653198 | 324.2253113 | -0,693481386 | 0,466370672 | -0,953166783 | 1,180277467 |
| 192 | 381.6293945 | 134.1097717 | -0,589874983 | 0,328733444 | -0,98967129 | 1,250812769 |
| 193 | 584.763916 | 134.1085968 | -0,688247681 | 0,554057241 | -0,984173596 | 1,118363976 |
| 194 | 375.9146423 | 215.025589 | -0,755117834 | 0,774776936 | -0,966209352 | 0,94655019 |
| 195 | 501.4121094 | 562.319397 | -0,670227826 | 0,59080416 | -1,008761168 | 1,088184834 |
| 196 | 795.8416138 | 762.3283691 | -0,725825906 | 0,787995637 | -0,992933869 | 0,930764139 |
| 197 | 566.0519409 | 458.1299744 | -0,465065807 | 0,283362687 | -1,068223834 | 1,249926925 |
| 198 | 751.4363403 | 202.1719208 | -0,396310985 | 0,257996351 | -1,107025027 | 1,245339751 |
| 199 | 496.3681641 | 394.1825256 | -0,563246369 | 0,675601661 | -1,109743953 | 0,997388721 |
| 200 | 814.1848755 | 326.182373 | -0,456625134 | 0,450598091 | -1,134623647 | 1,140650749 |
| 201 | 959.7723999 | 658.423645 | -0,502945244 | 0,771164775 | -1,162793398 | 0,894573927 |
| 202 | 381.1524963 | 218.0916443 | -0,418098331 | 0,61090982 | -1,199445844 | 1,006634355 |
| 203 | 375.0472107 | 360.0775146 | -0,329113871 | 0,479063779 | -1,22626543 | 1,076315522 |
| 204 | 496.3681641 | 467.1677856 | -0,138506308 | 0,065067969 | -1,182683468 | 1,256121755 |
| 205 | 381.3911438 | 197.1133881 | -0,442338526 | 0,758197963 | -1,201861858 | 0,886002481 |
| 206 | 678.2316895 | 435.1288452 | -0,238146499 | 0,449426115 | -1,271612167 | 1,060332537 |
| 207 | 381.1524963 | 414.2042542 | -0,329285115 | 0,730092347 | -1,267693043 | 0,866885781 |
| 208 | 1032.088623 | 370.3211365 | -0,277269244 | 0,627546132 | -1,285985112 | 0,935708225 |
| 209 | 559.052124 | 192.112442 | -0,192087665 | 0,564740241 | -1,320948601 | 0,94829607 |
| 210 | 1113.735718 | 302.2237549 | -0,3541421 | 0,938461542 | -1,246926308 | 0,662606895 |
| 211 | 710.8662109 | 325.1692505 | -0,403260917 | 1,103577495 | -1,179096103 | 0,478779495 |
| 212 | 1240.698853 | 363.3897705 | 0,067258142 | 0,097587526 | -1,30151391 | 1,136668205 |
| 213 | 678.1523438 | 434.1230774 | -0,180981815 | 0,668874323 | -1,33958149 | 0,851689041 |
| 214 | 1196.120239 | 312.1635742 | -0,083168842 | 1,121527314 | -1,292537332 | 0,254178822 |
| 215 | 1251.634033 | 793.0262451 | 0,438732028 | 0,132082835 | -1,431521177 | 0,860706329 |
| 216 | 1143.055664 | 521.4771729 | 0,57745415 | 0,126205534 | -1,447970867 | 0,744311214 |
| 217 | 1179.272949 | 170.0967102 | 0,480687946 | 0,451552987 | -1,498230577 | 0,565989614 |
| 218 | 496.9617004 | 228.0988617 | 0,98657763 | -0,287991136 | -1,271170139 | 0,572583675 |
| 219 | 1165.506836 | 307.2905884 | 0,515607953 | 0,879572809 | -1,40016973 | 0,004988999 |
| 220 | 1253.53772 | 458.2694397 | 0,737040699 | 0,39076668 | -1,477166414 | 0,349359036 |
| 221 | 874.9356689 | 182.0755005 | 0,76443249 | 0,510230184 | -1,456759691 | 0,182096973 |
| 222 | 744.8881836 | 849.6317139 | 0,583217144 | 0,922463656 | -1,33395195 | -0,17172879 |
| 223 | 1183.288696 | 473.7913208 | 0,812862098 | 0,666411102 | -1,372497916 | -0,106775351 |
| 224 | 535.0239258 | 678.5065918 | 0,756018043 | 0,847386837 | -1,262635589 | -0,340769261 |

| **B** |  |  |  |  |  |  |
| --- | --- | --- | --- | --- | --- | --- |
| **id** | **rtmed** | **mzmed (corrected)** | **Cont** | **Inf** | **Hx** | **Hxinf** |
| 1 | 743.257019 | 849.2285767 | 0,165950075 | 1,34046495 | -0,896335304 | -0,610079765 |
| 2 | 445.4208069 | 640.1903076 | -0,813996494 | 1,458616614 | -0,354558587 | -0,290061504 |
| 3 | 984.7740479 | 251.1499176 | -0,22303243 | 1,434091091 | -0,322414905 | -0,888643801 |
| 4 | 1150.30542 | 716.1467896 | -0,951204717 | 1,409249425 | -0,275515884 | -0,182528824 |
| 5 | 1104.879639 | 267.0377197 | 0,332635194 | 1,118411899 | -0,184349045 | -1,266698122 |
| 6 | 505.6720886 | 374.1912231 | 0,62241137 | 0,933732331 | -0,263311595 | -1,292832136 |
| 7 | 453.0568542 | 598.1531982 | -0,739962101 | 1,301522851 | 0,267316014 | -0,828876674 |
| 8 | 1086.420898 | 542.366394 | -0,278856278 | 1,185203791 | 0,290734172 | -1,197081804 |
| 9 | 248.1300964 | 342.0912476 | -0,584601879 | 1,246825457 | 0,333868235 | -0,996091843 |
| 10 | 230.9600677 | 354.082428 | 0,374244183 | 0,879295111 | 0,179797515 | -1,433336735 |
| 11 | 511.2563477 | 538.1327515 | 0,094529472 | 0,888025165 | 0,436022192 | -1,418576837 |
| 12 | 594.4488525 | 260.1278381 | -0,779921293 | 0,357006103 | 1,254252076 | -0,831336916 |
| 13 | 439.9689331 | 565.1806641 | -0,596580148 | 0,467678577 | 1,163782597 | -1,034881115 |
| 14 | 439.2344055 | 541.1616821 | -0,647958159 | 0,39115715 | 1,222514987 | -0,965713978 |
| 15 | 1255.330933 | 384.3278503 | -0,163135842 | 0,417663276 | 1,048863769 | -1,303391218 |
| 16 | 765.8433228 | 224.1391907 | -0,433695853 | 0,15178375 | 1,313374996 | -1,031462908 |
| 17 | 590.4743652 | 524.1817017 | -0,13799037 | 0,219481528 | 1,169515967 | -1,251007199 |
| 18 | 370.9425049 | 481.1273193 | 0,308980197 | 0,537043095 | 0,639588535 | -1,485611796 |
| 19 | 735.9400635 | 663.3237305 | 0,193135232 | 0,084890559 | 1,068638325 | -1,34666419 |
| 20 | 750.963501 | 440.2427979 | -0,168246746 | -0,125417188 | 1,3536551 | -1,059991241 |
| 21 | 771.6477661 | 703.3920288 | 0,073480032 | -0,034190733 | 1,203601003 | -1,242890358 |
| 22 | 668.2745361 | 552.1934814 | -0,027369212 | -0,08141949 | 1,276422262 | -1,167633533 |
| 23 | 450.9660339 | 504.1889954 | -0,083792277 | -0,217136055 | 1,354738235 | -1,053809881 |
| 24 | 594.6165771 | 267.0429077 | 0,310980052 | -0,043336283 | 1,063173652 | -1,330817461 |
| 25 | 749.2877197 | 826.2998047 | 0,401856363 | -0,130539566 | 1,044312835 | -1,315629721 |
| 26 | 478.7516479 | 779.4055176 | 0,656648457 | -0,00527891 | 0,759820581 | -1,411190152 |
| 27 | 1290.717285 | 494.3700256 | 0,133774251 | -0,331642419 | 1,293199897 | -1,095331788 |
| 28 | 955.4966431 | 169.9938354 | 0,009298545 | -0,45047456 | 1,382411599 | -0,941235602 |
| 29 | 734.6693726 | 280.1281433 | 0,310358673 | -0,449115068 | 1,229625225 | -1,090868831 |
| 30 | 715.0662842 | 244.1644287 | 0,401753515 | -0,464000672 | 1,175972939 | -1,113725781 |
| 31 | 547.3361206 | 296.1213379 | 0,451888621 | -0,484324306 | 1,147743464 | -1,115307808 |
| 32 | 545.0895996 | 478.250885 | 0,134131715 | -0,831860423 | 1,36044836 | -0,662719607 |
| 33 | 546.6357422 | 456.2689819 | 0,281180501 | -0,953918636 | 1,28100884 | -0,608270645 |
| 34 | 690.8051147 | 604.1845703 | 0,343114168 | -0,89972806 | 1,25755477 | -0,700940847 |
| 35 | 950.7770996 | 698.1107178 | 0,367581248 | -0,908551037 | 1,243391633 | -0,702421784 |
| 36 | 543.9458618 | 906.6487427 | 0,381422579 | -0,883372963 | 1,237938166 | -0,735987783 |
| 37 | 490.2504578 | 276.0989075 | 0,728899479 | -1,194320917 | 0,91608268 | -0,450661242 |
| 38 | 581.2160645 | 208.1078796 | 0,431442261 | -0,819747567 | 1,210636973 | -0,822331727 |
| 39 | 578.0338135 | 854.5881348 | 0,788267255 | -1,166261315 | 0,877121151 | -0,49912715 |
| 40 | 734.980896 | 212.1387939 | 0,460156828 | -0,787993193 | 1,191922069 | -0,864085734 |
| 41 | 924.9797363 | 292.2016602 | 0,366899967 | -0,674479067 | 1,240794539 | -0,933215499 |
| 42 | 706.6130981 | 892.5005493 | 0,719680905 | -0,985370934 | 0,991858363 | -0,726168335 |
| 43 | 647.1469727 | 198.1251373 | 0,612593889 | -0,86970073 | 1,086493254 | -0,829386413 |
| 44 | 579.0178833 | 1005.58606 | 0,754371285 | -1,016821027 | 0,956621409 | -0,694171667 |
| 45 | 1120.701294 | 169.9934387 | 0,658399761 | -0,886492193 | 1,050696731 | -0,822604239 |
| 46 | 545.1564331 | 228.1335754 | 0,547073603 | -0,757972181 | 1,131107569 | -0,920208871 |
| 47 | 581.9677734 | 1035.731689 | 0,560518384 | -0,739100397 | 1,119728804 | -0,941146731 |
| 48 | 545.6436157 | 229.1377106 | 0,590939701 | -0,749853551 | 1,098251104 | -0,939337254 |
| 49 | 545.0895996 | 210.1230927 | 0,678380549 | -0,773569048 | 1,03168273 | -0,936494291 |
| 50 | 543.9458618 | 906.6428223 | 0,617000997 | -0,712894797 | 1,07422483 | -0,978331089 |
| 51 | 543.9458618 | 906.6465454 | 0,634081066 | -0,718404651 | 1,06154418 | -0,977220535 |
| 52 | 789.2235107 | 718.2489014 | 0,830104411 | -0,871860802 | 0,901194453 | -0,859438121 |
| 53 | 765.5392456 | 704.2333374 | 0,846641481 | -0,863176405 | 0,885190487 | -0,868655562 |
| 54 | 687.0768433 | 211.1265259 | 0,785519779 | -0,79347831 | 0,940280259 | -0,932321787 |
| 55 | 545.5874634 | 211.1277924 | 0,72174257 | -0,707680643 | 0,987498283 | -1,001560211 |
| 56 | 581.2160645 | 1036.731567 | 0,677539229 | -0,624951839 | 1,010285616 | -1,062872887 |
| 57 | 890.1254883 | 215.036377 | 0,994176745 | -0,927749515 | 0,724640966 | -0,791068196 |
| 58 | 686.2174683 | 210.1230621 | 0,75246203 | -0,647296906 | 0,949734211 | -1,054899335 |
| 59 | 880.6430054 | 846.4447021 | 0,717745066 | -0,459821522 | 0,929372728 | -1,187296271 |
| 60 | 535.380249 | 1156.616699 | 0,895822942 | -0,462848425 | 0,755505323 | -1,188479781 |
| 61 | 477.6163025 | 663.3892822 | 0,927693725 | 0,46416682 | -0,004809579 | -1,387050986 |
| 62 | 400.7694397 | 480.1242065 | 0,919342458 | -0,100059383 | 0,541459203 | -1,360742211 |
| 63 | 869.4173584 | 430.1307068 | 0,941491127 | 0,485586524 | -0,057976138 | -1,369101524 |
| 64 | 445.1203613 | 498.0929565 | 0,814659417 | 0,712121964 | -0,187334508 | -1,339446902 |
| 65 | 1190.393188 | 637.4931641 | 0,972738802 | -0,232418895 | 0,558743656 | -1,299063563 |
| 66 | 465.5814514 | 434.1097107 | 1,05970192 | -0,317628652 | 0,495221883 | -1,237295151 |
| 67 | 1129.649536 | 146.0514679 | 1,199935436 | -0,075877912 | 0,117178403 | -1,241235971 |
| 68 | 1260.292603 | 656.4484253 | 1,49966836 | -0,525399923 | -0,500368416 | -0,47389999 |
| 69 | 893.8615723 | 432.1920471 | 0,525279701 | 0,332160831 | -1,488472223 | 0,631031692 |
| 70 | 1297.393433 | 615.5131836 | 0,848937571 | -0,12438754 | -1,362611532 | 0,638061523 |
| 71 | 958.6333008 | 601.3966675 | 1,181446552 | -0,357655406 | -1,16586864 | 0,342077553 |
| 72 | 1164.646606 | 837.5964355 | 1,266548634 | -0,812935412 | -0,788993478 | 0,335380316 |
| 73 | 916.2926636 | 837.597229 | 0,657690466 | -1,038724542 | -0,650880992 | 1,031915069 |
| 74 | 537.6186523 | 945.5878906 | 0,234464005 | -0,964702308 | -0,569694221 | 1,29993248 |
| 75 | 876.4979248 | 837.5985718 | 0,039092273 | -0,858439267 | -0,571623862 | 1,390970826 |
| 76 | 811.6781006 | 438.1445618 | 0,650032759 | -1,303822994 | -0,249204859 | 0,90299505 |
| 77 | 1249.450317 | 322.2197876 | 0,781527817 | -1,402065039 | -0,029506898 | 0,650044084 |
| 78 | 1154.668213 | 718.1911011 | 0,135017768 | -1,264301181 | 1,175244808 | -0,045961473 |
| 79 | 643.8092651 | 240.170578 | 0,319066078 | -1,488200188 | 0,58141017 | 0,587723911 |
| 80 | 807.0216675 | 718.4857788 | 0,162175715 | -1,450846195 | 0,782914519 | 0,505756021 |
| 81 | 611.5649414 | 950.6837769 | 0,121132746 | -1,44649899 | 0,748327375 | 0,577038825 |
| 82 | 405.8056335 | 491.3140869 | -0,478017658 | -1,134390712 | 1,110117435 | 0,502290845 |
| 83 | 535.6951294 | 678.5053711 | -0,327079028 | -1,266218662 | 0,695626378 | 0,897671282 |
| 84 | 525.2180176 | 603.3967285 | -0,778622925 | -0,943102419 | 0,966065109 | 0,755660236 |
| 85 | 1209.3302 | 500.3706665 | -1,047409296 | -0,621038318 | 1,084609866 | 0,583837748 |
| 86 | 1248.593506 | 369.3674927 | -0,789116681 | -0,876415014 | 0,499454022 | 1,166077733 |
| 87 | 640.7641602 | 1056.743896 | -1,189023018 | -0,428254813 | 0,590661526 | 1,026616335 |
| 88 | 851.8951416 | 724.5098877 | -1,126085281 | -0,488913953 | 0,50026685 | 1,114732385 |
| 89 | 296.6820068 | 288.0828552 | -1,165463209 | -0,43627137 | 0,510534883 | 1,091199756 |
| 90 | 694.1434326 | 724.512146 | -0,823507428 | -0,740932465 | 0,258235872 | 1,306203961 |
| 91 | 777.8493652 | 715.4857178 | -0,956185103 | -0,424605608 | 0,000421234 | 1,380369544 |
| 92 | 948.5161133 | 1054.744385 | -0,74708885 | -0,355020344 | -0,373200923 | 1,475310087 |
| 93 | 446.7094727 | 242.1490936 | -0,854218543 | -0,227123365 | -0,363230437 | 1,44457233 |
| 94 | 1014.788452 | 1063.74585 | -0,941544056 | -0,130935803 | -0,335896522 | 1,408376455 |
| 95 | 811.0167847 | 356.2148132 | -1,113422275 | -0,000758794 | -0,197757393 | 1,311938405 |
| 96 | 778.0568848 | 601.3978882 | -1,35264051 | 0,171409175 | 0,119803205 | 1,061428189 |
| 97 | 481.2229004 | 242.1496735 | -1,033213258 | 0,011428624 | -0,328845143 | 1,350629687 |
| 98 | 414.6809692 | 240.1339264 | -0,903149068 | 0,036088042 | -0,517027736 | 1,384088755 |
| 99 | 812.3544312 | 853.6600952 | -1,117586493 | 0,238386899 | -0,369002819 | 1,248202324 |
| 100 | 481.6550903 | 243.1501923 | -0,92802304 | 0,190721184 | -0,588060021 | 1,325361848 |
| 101 | 337.0310669 | 164.0444641 | -1,082447648 | 0,321739465 | -0,466833174 | 1,227541327 |
| 102 | 652.7540894 | 249.1338196 | -1,126799941 | 0,387891084 | -0,438898623 | 1,17780745 |
| 103 | 657.2955933 | 200.1392975 | -0,714462042 | 0,197324768 | -0,817742527 | 1,334879756 |
| 104 | 812.3572388 | 966.734375 | -1,097141623 | 0,422750294 | -0,496519178 | 1,170910478 |
| 105 | 337.3730164 | 350.0743713 | -0,704855979 | 0,21316573 | -0,835093975 | 1,326784253 |
| 106 | 831.2740479 | 314.1984253 | -0,976508141 | 0,37533465 | -0,628081918 | 1,229255438 |
| 107 | 956.2185669 | 724.5115967 | -0,278776556 | 4,22E-05 | -1,061348677 | 1,340083003 |
| 108 | 812.1209106 | 289.2324524 | -1,08319509 | 0,44454661 | -0,524811685 | 1,163460135 |
| 109 | 791.9085693 | 328.2229919 | -1,282906413 | 0,558847308 | -0,262354106 | 0,98641324 |
| 110 | 583.0664673 | 1012.703735 | -1,202385664 | 0,574407399 | -0,402651161 | 1,030629396 |
| 111 | 492.913147 | 302.1065979 | -0,946381152 | 0,443571717 | -0,69231087 | 1,195120335 |
| 112 | 812.0597534 | 288.2279968 | -0,983352661 | 0,501288593 | -0,671528697 | 1,153592825 |
| 113 | 109.6608887 | 154.0241699 | -1,103098035 | 0,659103692 | -0,568632782 | 1,012627125 |
| 114 | 355.8948669 | 195.0583496 | -1,272556663 | 0,800927997 | -0,324496806 | 0,796125472 |
| 115 | 221.7764435 | 152.0450134 | -0,250113279 | 0,248935908 | -1,19846797 | 1,199645281 |
| 116 | 356.9264832 | 145.0498199 | -0,836319983 | 0,581607878 | -0,854952931 | 1,109665036 |
| 117 | 356.1373596 | 194.0553894 | -1,119496465 | 0,764672875 | -0,563888848 | 0,918712378 |
| 118 | 328.8818054 | 193.0713654 | -1,093326449 | 0,75573349 | -0,598184466 | 0,935777426 |
| 119 | 375.1867371 | 136.050293 | -0,890268564 | 0,652311146 | -0,817397356 | 1,055354714 |
| 120 | 811.4549561 | 290.2361145 | -0,829749286 | 0,657759905 | -0,879426897 | 1,051416278 |
| 121 | 811.6781006 | 324.2020874 | -0,975134552 | 0,753098369 | -0,742593467 | 0,96462965 |
| 122 | 414.1376648 | 196.1417084 | -0,860507071 | 0,704339683 | -0,857661784 | 1,013829112 |
| 123 | 722.9172974 | 255.1537933 | -0,250483871 | 0,404996365 | -1,252266645 | 1,097754121 |
| 124 | 577.3856812 | 830.5717773 | -0,939698994 | 0,863720298 | -0,789073706 | 0,865052402 |
| 125 | 828.7459717 | 240.170578 | -0,794286013 | 0,806867421 | -0,933112025 | 0,920530617 |
| 126 | 539.8518677 | 816.5591431 | -0,145258531 | 0,463921309 | -1,323999524 | 1,005336761 |
| 127 | 564.2888794 | 297.1356201 | -0,933609963 | 0,917858541 | -0,793929815 | 0,809681237 |
| 128 | 442.1629639 | 313.132019 | -0,945383489 | 1,108765364 | -0,738783956 | 0,575402081 |
| 129 | 388.4477234 | 254.1139832 | -0,723544717 | 1,101924896 | -0,960765958 | 0,582385838 |
| 130 | 525.25 | 249.0993042 | -0,701253593 | 1,182522893 | -0,945642829 | 0,464373499 |
| 131 | 253.0501556 | 165.0411377 | -0,835825384 | 1,288117647 | -0,745696306 | 0,293404013 |
