# Supplementary material for: Priming by Hexanoic Acid Induce Activation of Mevalonic and Linolenic Pathways and Promotes the Emission of Plant Volatiles
Source: Front Plant Sci. 2016 Apr 12;7:495. doi: 10.3389/fpls.2016.00495 (PMC4828442; doi:10.3389/fpls.2016.00495)
Supplement: Supplementary file 3 [file Table_3.DOCX]

Supplementary Material

Priming by Hexanoic acid induce activation of mevalonic and linolenic pathways and promotes the emission of plant volatiles.

Eugenio Llorens*, Gemma Camañes, Leonor Lapeña, Pilar García-Agustín

*** Correspondence:** Dr. Eugenio Llorens: ellorens@uji.es

# Supplementary Table 3: List of detected compounds in the GS-MS analysis

| **Compound** | **Detected** | **Compound** | **Detected** |
| --- | --- | --- | --- |
| **Camphor** | + | **6-Methyl-5-hepten-2-one** | + |
| **E-2-HexenylAcetate** | + | **Trans-2-hexen-1-al** | + |
| **3-Carene** | + | **Methyl salicilate** | + |
| **Alfa Pinene** | + | **Hexanal** | - |
| **Terpineol** | + | **Guaiacol** | + |
| **3-Methyltiopropionaldehyde** | - | **Ethyl salicilate** | + |
| **E-2-Hexen-1-ol** | - | **2-Mehtyl-1-butanol** | - |
| **1-hexanol** | + | **Trans,trans-2,4-hexadienal** | - |
| **Isoamyl Acetate** | - | **Salicilaldehyde** | - |
| **Gamma Terpinene** | + | **1-Octanol** | + |
| **E-2-Octenal** | - | **Trans,trans-2,4-heptadienal** | - |
| **Z-3-hexen-1-ol** | + | **Trans,trans-2,4-decadienal** | + |
| **R-Limonene** | + | **Beta-cyclocitral** | - |
| **Citral** | + | **2-Octanone** | - |
| **Geranyl Acetone** | + | **2-Heptanone** | - |
| **Nonanal** | - | **Benzaldehyde** | + |
| **2-Isobutylthiazole** | - | **Butyl acetate** | + |
| **2-Carene** | + | **2,6-Dimethyl-6-hepten-2-ol** | - |
| **E-2-heptenal** | + | **4-Methoxyphenol** | - |
| **6-methyl-5-hepten-2-ol** | + | **Diphenyl-ether** | + |
| **Beta-Ionone** | + | **Methyl-jasmonate** | - |
| **Eugenol** | - | **Methyl-dihydrojasmonate** | - |
| **Linalool** | + | **Damascenone** | - |
| **Phenethyl Alcohol** | + |  |  |
