# Supplementary material for: Priming by Hexanoic Acid Induce Activation of Mevalonic and Linolenic Pathways and Promotes the Emission of Plant Volatiles
Source: Front Plant Sci. 2016 Apr 12;7:495. doi: 10.3389/fpls.2016.00495 (PMC4828442; doi:10.3389/fpls.2016.00495)
Supplement: Supplementary file 4 [file Image_1.PDF]

Supplementary figure 1: Compounds putatively identified in pathways in negative ionization. Red dots represent compounds identified by exact mass in the linolenic acid metabolism, terpenoid backbone biosynthesis and diterpenoid biosynthesis pathways.

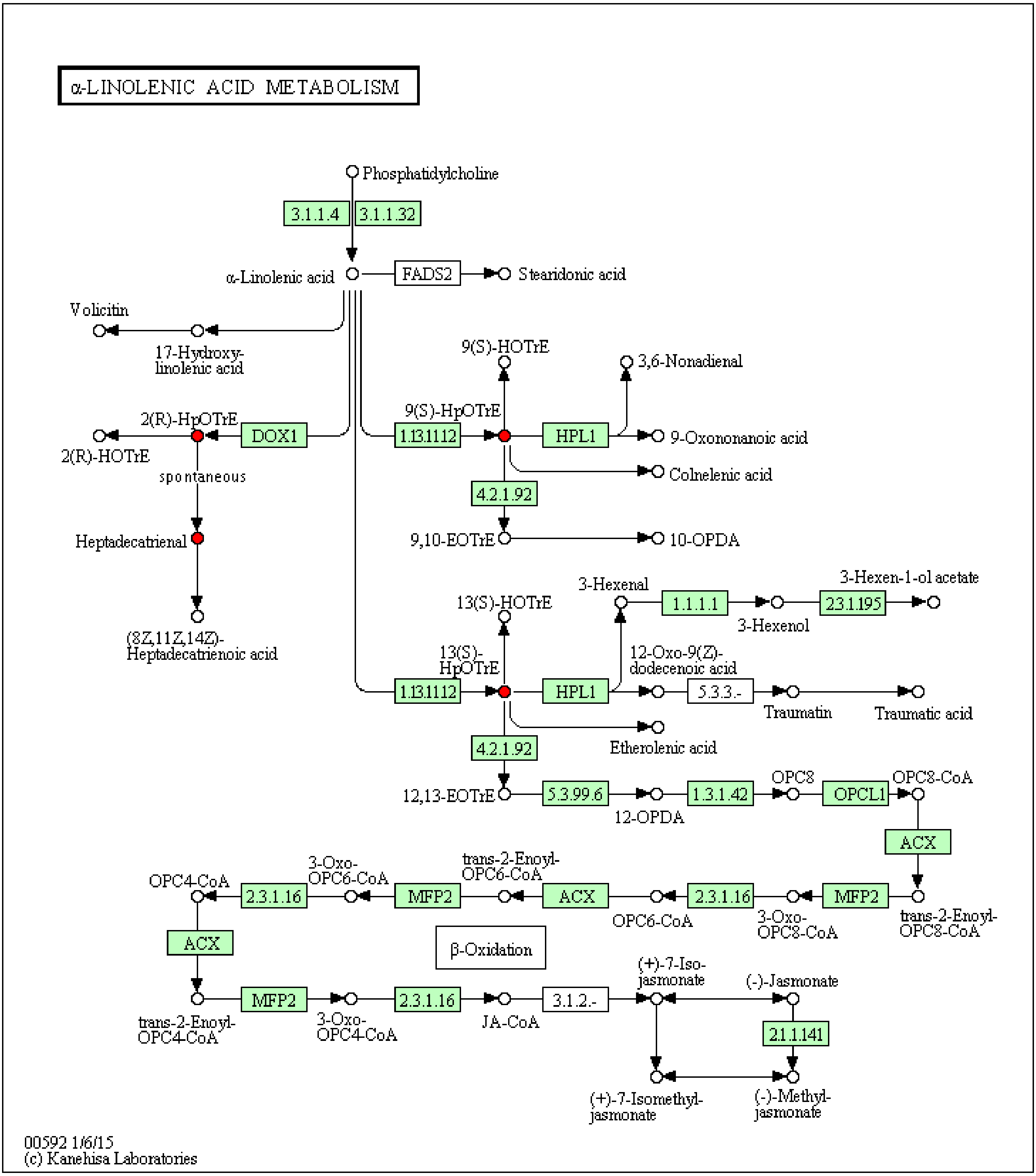

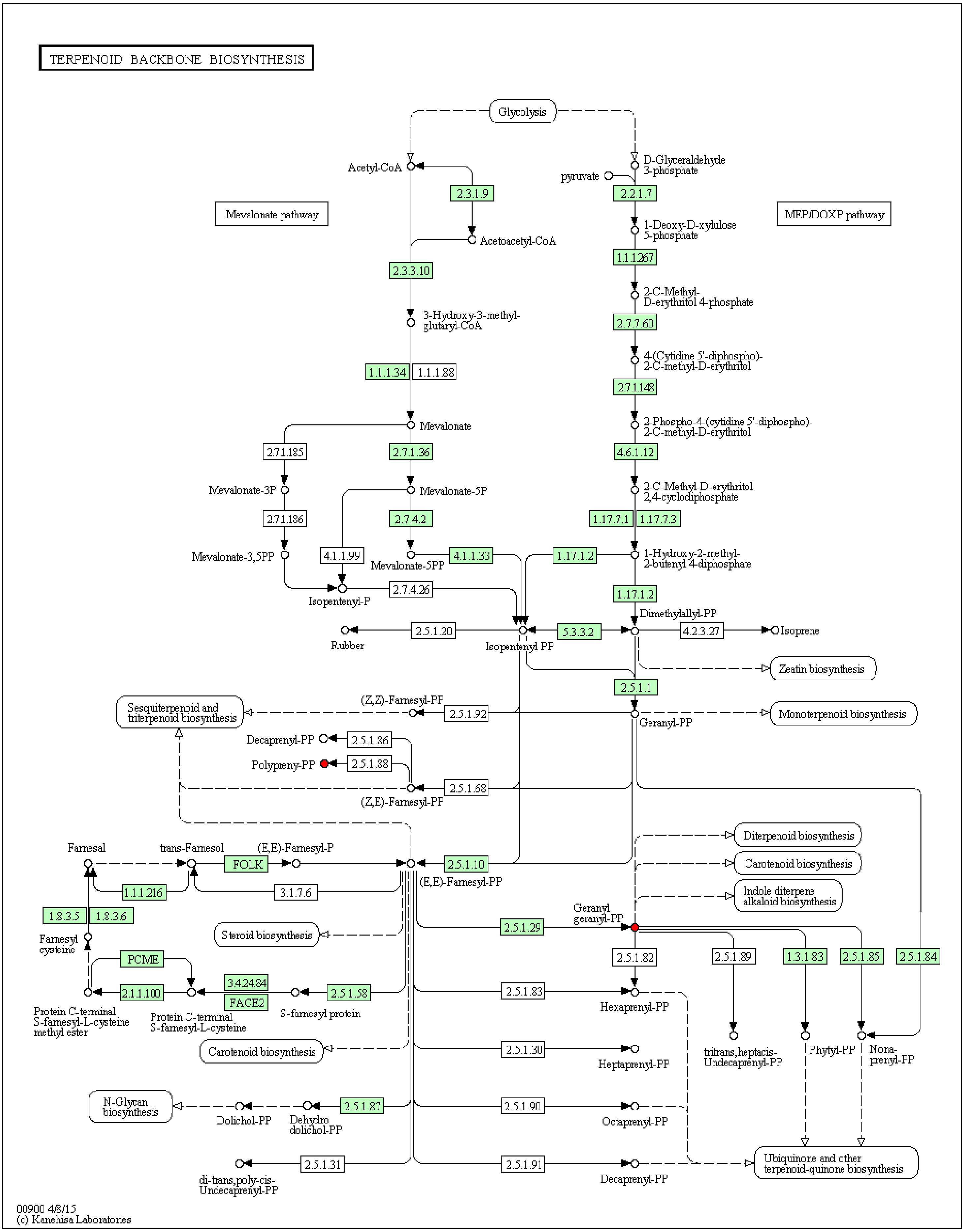
TERPENOID BACKBONE BIOSYNTHESIS
Glycolysis
Acetyl-CoA
D-Glyceraldehyde 3-phosphate
pyruvate
2.3.1.9
2.2.1.7
Mevalonate pathway
MEP/DOXP pathway
Acetoacetyl-CoA
1-Deoxy-D-xylulose 5-phosphate
2.3.3.10
1.1.1.267
2-C-Methyl-D-erythritol 4-phosphate
3-Hydroxy-3-methyl-glutaryl-CoA
2.7.7.60
4-(Cytidine 5'-diphospho)-2-C-methyl-D-erythritol
1.1.1.34
1.1.1.88
2.7.1.148
Mevalonate
2-Phospho-4-(cytidine 5'-diphospho)-2-C-methyl-D-erythritol
2.7.1.185
2.7.1.36
4.6.1.12
Mevalonate-3P
Mevalonate-5P
2-C-Methyl-D-erythritol 2,4-cyclodiphosphate
2.7.1.186
2.7.4.2
1.17.7.1
1.17.7.3
Mevalonate-3,5PP
4.1.1.99
Mevalonate-5PP
4.1.1.33
1.17.1.2
1-Hydroxy-2-methyl-2-butenyl 4-diphosphate
Isopentenyl-P
2.7.4.26
1.17.1.2
Dimethylallyl-PP
Rubber
2.5.1.20
Isopentenyl-PP
5.3.3.2
4.2.3.27
Isoprene
Zeatin biosynthesis
2.5.1.1
Sesquiterpenoid and triterpenoid biosynthesis
(Z,Z)-Farnesyl-PP
2.5.1.92
Geranyl-PP
Monoterpenoid biosynthesis
Decaprenyl-PP
2.5.1.86
Polypreny-PP
2.5.1.88
2.5.1.68
(Z,E)-Farnesyl-PP
Diterpenoid biosynthesis
Farnesal
trans-Farnesol
(E,E)-Farnesyl-P
FOLK
2.5.1.10
Carotenoid biosynthesis
(E,E)-Farnesyl-PP
1.1.1.216
3.1.7.6
Indole diterpene alkaloid biosynthesis
1.8.3.5
1.8.3.6
Geranyl geranyl-PP
Steroid biosynthesis
2.5.1.29
Farnesyl cysteine
PCME
2.5.1.82
2.5.1.89
1.3.1.83
2.5.1.85
2.5.1.84
3.4.24.84
2.1.1.100
FACE2
2.5.1.58
2.5.1.83
Protein C-terminal S-farnesyl-L-cysteine methyl ester
Protein C-terminal S-farnesyl-L-cysteine
S-farnesyl protein
Hexaprenyl-PP
tritrans,heptacis-Undecaprenyl-PP
Phytyl-PP
Nona-prenyl-PP
Carotenoid biosynthesis
2.5.1.30
Heptaprenyl-PP
N-Glycan biosynthesis
Dolichol-PP
Dehydro dolichol-PP
2.5.1.87
2.5.1.90
Octaprenyl-PP
2.5.1.31
2.5.1.91
Ubiquinone and other terpenoid-quinone biosynthesis
di-trans,poly-cis-Undecaprenyl-PP
Decaprenyl-PP
00900 4/8/15
(c) Kanehisa Laboratories

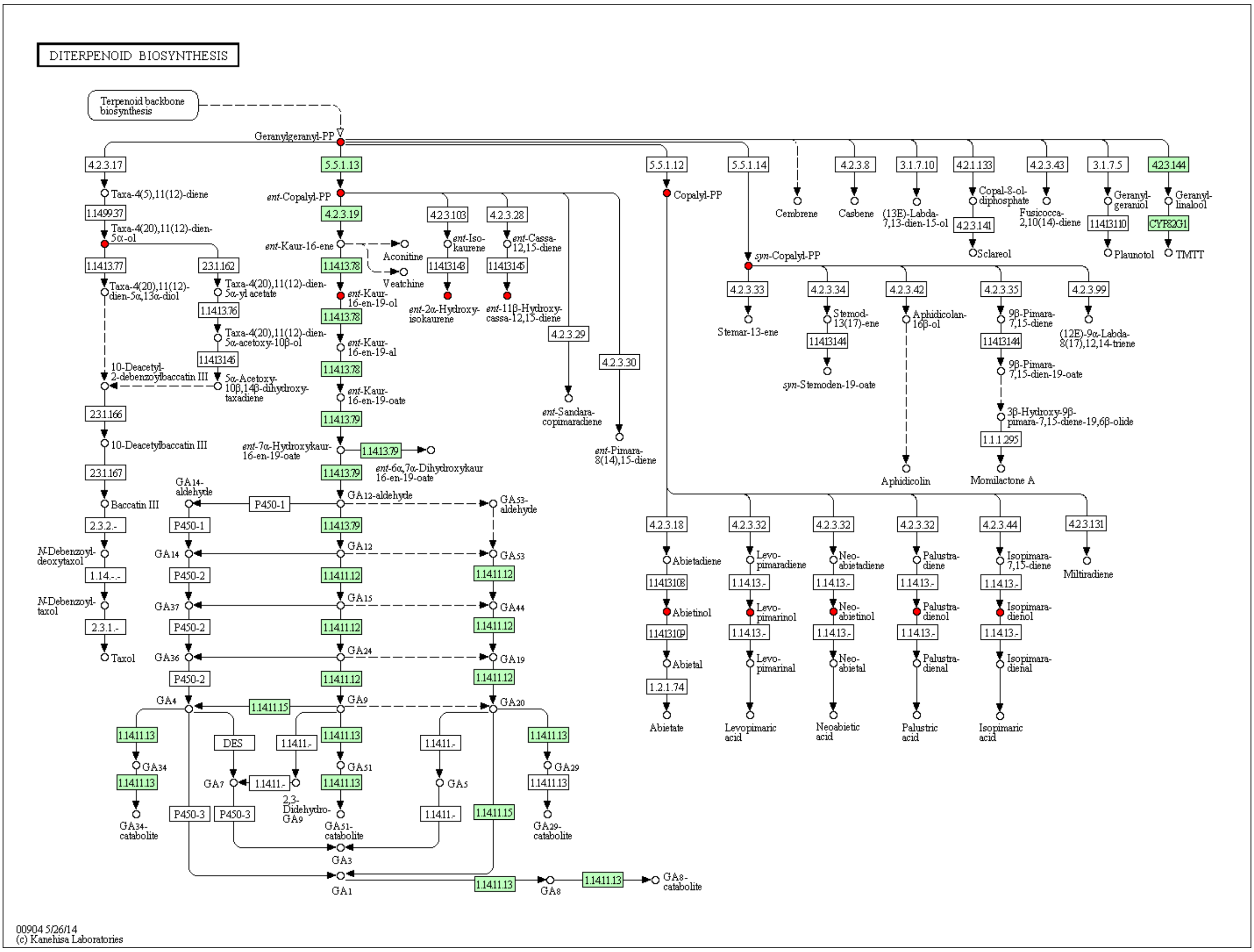
DITERPENOID BIOSYNTHESIS
Terpenoid backbone biosynthesis
Geranylgeranyl-PP
4.2.3.17
5.5.1.13
5.5.1.12
5.5.1.14
4.2.3.8
3.1.7.10
4.2.1.133
4.2.3.43
3.1.7.5
4.2.3.144
Taxa-4(5),11(12)-diene
1.14.99.37
Taxa-4(20),11(12)-dien-5α-ol
1.14.13.77
2.3.1.162
Taxa-4(20),11(12)-dien-5α,13α-diol
Taxa-4(20),11(12)-dien-5α-yl acetate
1.14.13.76
Taxa-4(20),11(12)-dien-5α-acetoxy-10β-ol
1.14.13.146
10-Deacetyl-2-debenzoylbaccatin III
5α-Acetoxy-10β,14β-dihydroxy-taxadiene
2.3.1.166
10-Deacetylbaccatin III
2.3.1.167
Baccatin III
2.3.2.-
N-Debenzoyl-deoxytaxol
1.14.-.-
N-Debenzoyl-taxol
2.3.1.-
Taxol
ent-Copalyl-PP
4.2.3.19
ent-Kaur-16-ene
Aconitine
Veatchine
1.14.13.78
ent-Kaur-16-en-19-ol
ent-Kaur-16-en-19-al
ent-Kaur-16-en-19-oate
1.14.13.79
ent-7α-Hydroxykaur-16-en-19-oate
ent-6α,7α-Dihydroxykaur 16-en-19-oate
4.2.3.103
ent-Iso-kaurene
1.14.13.143
ent-2α-Hydroxy-isokaurene
4.2.3.28
ent-Cassa-12,15-diene
1.14.13.145
ent-11β-Hydroxy-cassa-12,15-diene
4.2.3.29
ent-Sandara-copimaradiene
4.2.3.30
ent-Pimara-8(14),15-diene
GA12-aldehyde
GA14-aldehyde
P450-1
GA53-aldehyde
GA12
GA14
GA53
P450-2
1.14.11.12
GA15
GA37
GA44
GA24
GA36
GA19
GA9
GA4
GA20
1.14.11.15
1.14.11.13
GA34
GA34-catabolite
DES
GA7
1.14.11.-
2,3-Didehydro-GA9
GA51
GA51-catabolite
GA5
GA29
GA29-catabolite
P450-3
GA3
GA1
GA8
GA8-catabolite
Copalyl-PP
Cembrene
Casbene
(13E)-Labda-7,13-dien-15-ol
Copal-8-ol-diphosphate
4.2.3.141
Sclareol
Fusicocca-2,10(14)-diene
Geranyl-geraniol
1.14.13.110
Plaunotol
Geranyl-linalool
CYP82G1
TMTT
syn-Copalyl-PP
4.2.3.33
Stemar-13-ene
4.2.3.34
Stemod-13(17)-ene
1.14.13.144
syn-Stemoden-19-oate
4.2.3.42
Aphidicolan-16β-ol
Aphidicolin
4.2.3.35
9β-Pimara-7,15-diene
9β-Pimara-7,15-dien-19-oate
3β-Hydroxy-9β-pimara-7,15-diene-19,6β-olide
1.1.1.295
Momilactone A
4.2.3.99
(12E)-9α-Labda-8(17),12,14-triene
4.2.3.18
Abietadiene
1.14.13.108
Abietinol
1.14.13.109
Abietal
1.2.1.74
Abietate
4.2.3.32
Levo-pimaradiene
1.14.13.-
Levo-pimarinol
Levo-pimarinal
Levopimaric acid
Neo-abietadiene
Neo-abietinol
Neo-abietal
Neoabietic acid
Palustra-diene
Palustra-dienol
Palustra-dienal
Palustric acid
4.2.3.44
Isopimara-7,15-diene
Isopimara-dienol
Isopimara-dienal
Isopimaric acid
4.2.3.131
Miltiradiene
00904 5/26/14
(c) Kanehisa Laboratories
