# Supplementary material for: Priming by Hexanoic Acid Induce Activation of Mevalonic and Linolenic Pathways and Promotes the Emission of Plant Volatiles
Source: Front Plant Sci. 2016 Apr 12;7:495. doi: 10.3389/fpls.2016.00495 (PMC4828442; doi:10.3389/fpls.2016.00495)
Supplement: Supplementary file 5 [file Image_2.PDF]

Supplementary figure 2: Compounds putatively identified in pathways in positive ionization. Red dots represent compounds identified by exact mass in the linolenic acid metabolism, terpenoid backbone biosynthesis, monoterpenoid biosynthesis, diterpenoid biosynthesis, limonene and pinene degradation, pentose phosphate and carbon metabolism pathways.

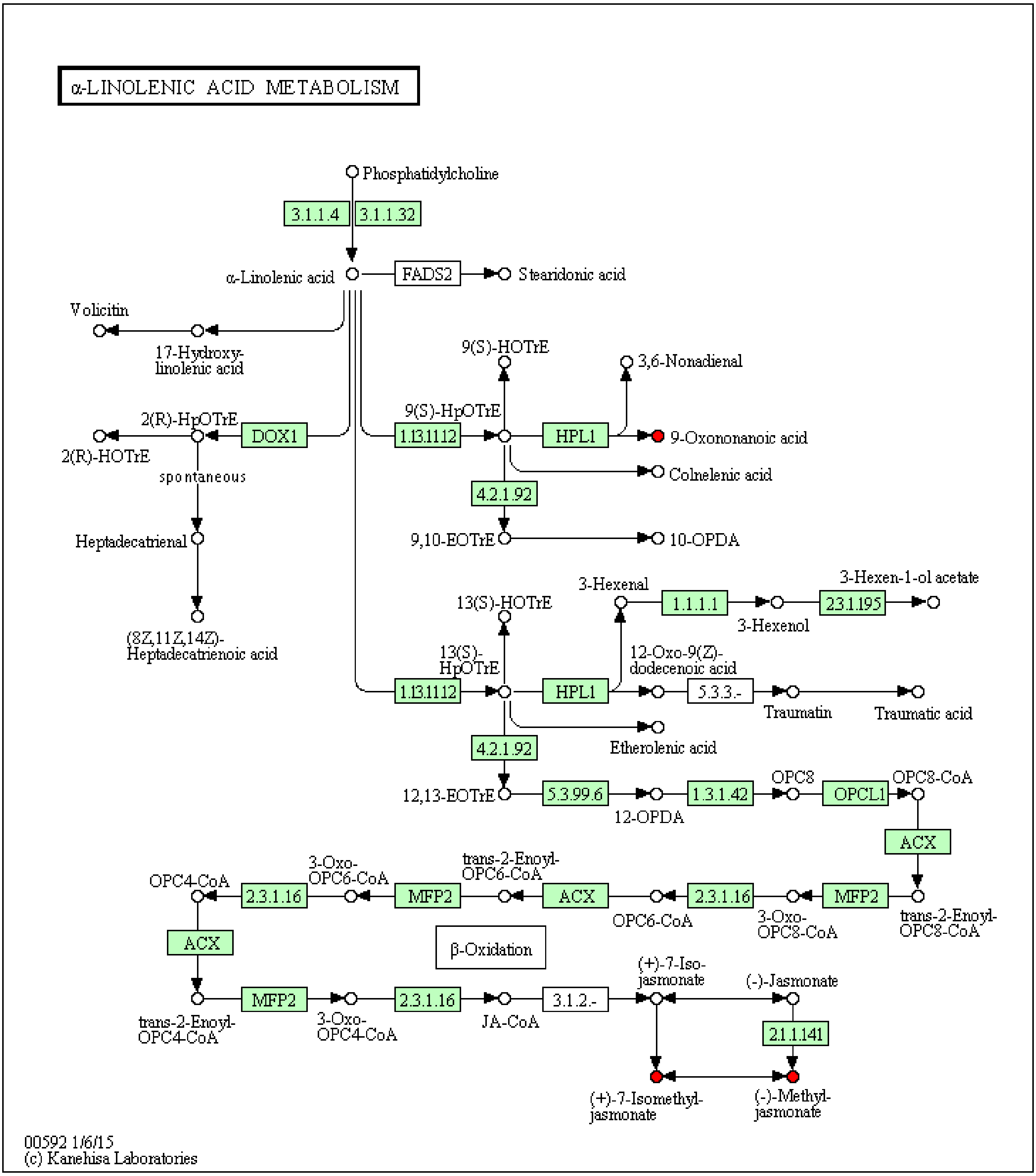

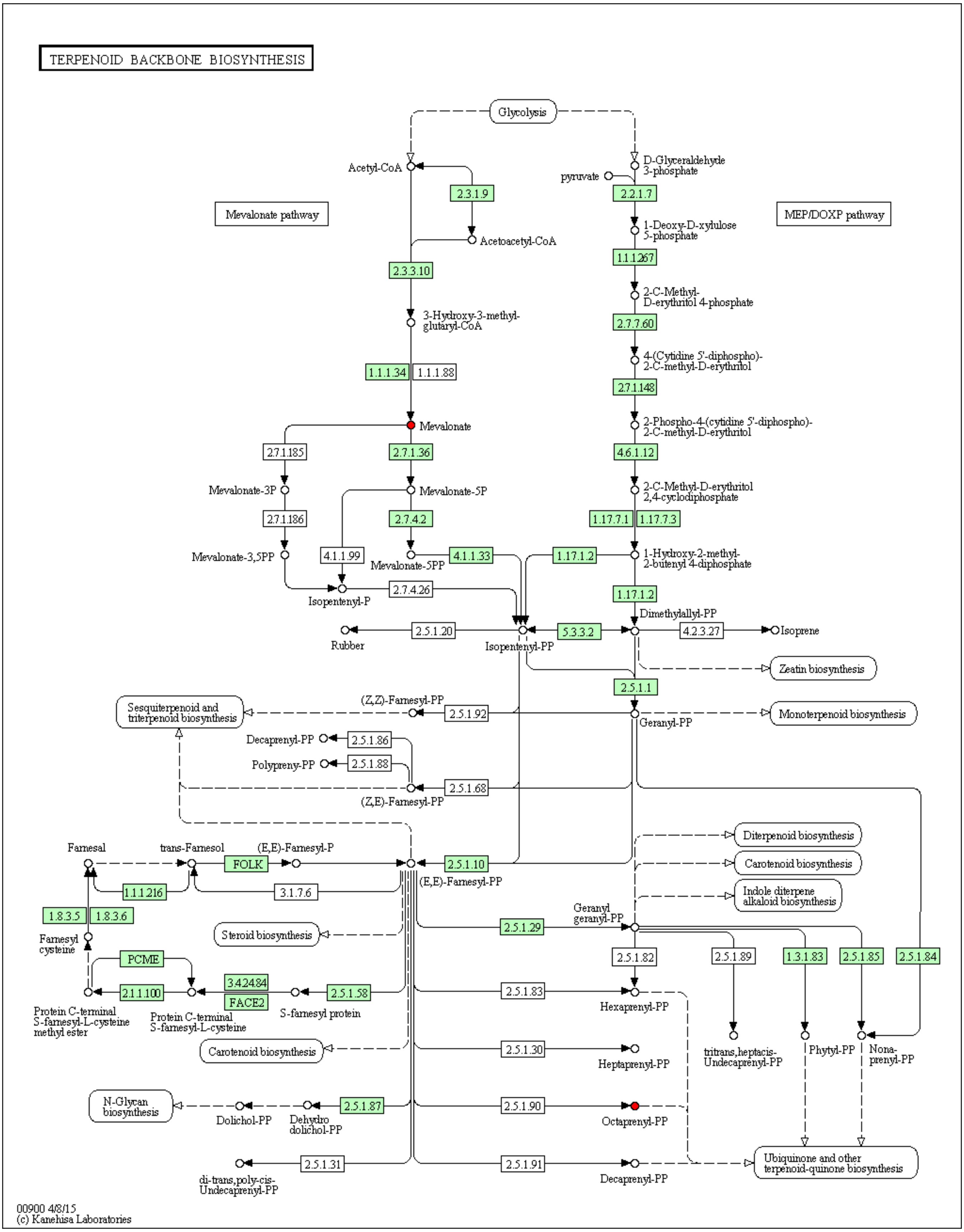
TERPENOID BACKBONE BIOSYNTHESIS
Glycolysis
Acetyl-CoA
2.3.1.9
Acetoacetyl-CoA
Mevalonate pathway
2.3.3.10
3-Hydroxy-3-methyl-glutaryl-CoA
1.1.1.34
1.1.1.88
Mevalonate
2.7.1.185
2.7.1.36
Mevalonate-3P
Mevalonate-5P
2.7.1.186
2.7.4.2
Mevalonate-3,5PP
4.1.1.99
Mevalonate-5PP
4.1.1.33
Isopentenyl-P
2.7.4.26
Rubber
2.5.1.20
Isopentenyl-PP
5.3.3.2
D-Glyceraldehyde 3-phosphate
pyruvate
2.2.1.7
MEP/DOXP pathway
1-Deoxy-D-xylulose 5-phosphate
1.1.1.267
2-C-Methyl-D-erythritol 4-phosphate
2.7.7.60
4-(Cytidine 5'-diphospho)-2-C-methyl-D-erythritol
2.7.1.148
2-Phospho-4-(cytidine 5'-diphospho)-2-C-methyl-D-erythritol
4.6.1.12
2-C-Methyl-D-erythritol 2,4-cyclodiphosphate
1.17.7.1
1.17.7.3
1.17.1.2
1-Hydroxy-2-methyl-2-butenyl 4-diphosphate
1.17.1.2
Dimethylallyl-PP
4.2.3.27
Isoprene
Zeatin biosynthesis
2.5.1.1
Sesquiterpenoid and triterpenoid biosynthesis
(Z,Z)-Farnesyl-PP
2.5.1.92
Geranyl-PP
Monoterpenoid biosynthesis
Decaprenyl-PP
2.5.1.86
Polypreny-PP
2.5.1.88
2.5.1.68
(Z,E)-Farnesyl-PP
Farnesal
trans-Farnesol
(E,E)-Farnesyl-P
FOLK
2.5.1.10
(E,E)-Farnesyl-PP
1.1.1.216
3.1.7.6
1.8.3.5
1.8.3.6
Farnesyl cysteine
PCME
Steroid biosynthesis
3.4.24.84
FACE2
2.1.1.100
2.5.1.58
S-farnesyl protein
Protein C-terminal S-farnesyl-L-cysteine methyl ester
Protein C-terminal S-farnesyl-L-cysteine
Carotenoid biosynthesis
Diterpenoid biosynthesis
Carotenoid biosynthesis
Indole diterpene alkaloid biosynthesis
2.5.1.29
Geranyl geranyl-PP
2.5.1.82
2.5.1.89
1.3.1.83
2.5.1.85
2.5.1.84
2.5.1.83
Hexaprenyl-PP
tritrans,heptacis-Undecaprenyl-PP
Phytyl-PP
Nona-prenyl-PP
2.5.1.30
Heptaprenyl-PP
N-Glycan biosynthesis
Dolichol-PP
Dehydro dolichol-PP
2.5.1.87
2.5.1.90
Octaprenyl-PP
Ubiquinone and other terpenoid-quinone biosynthesis
2.5.1.31
di-trans,poly-cis-Undecaprenyl-PP
2.5.1.91
Decaprenyl-PP
00900 4/8/15
(c) Kanehisa Laboratories

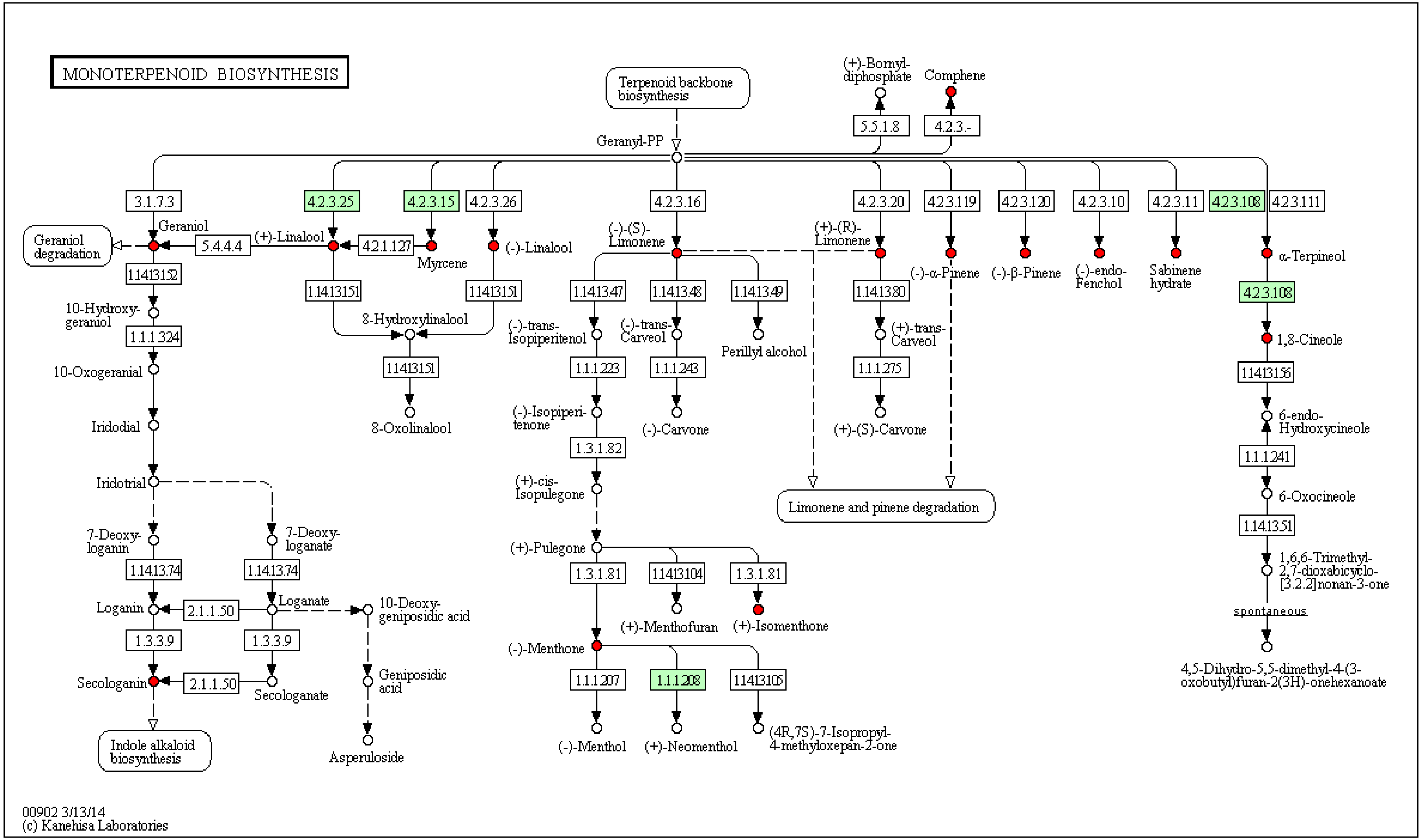
MONOTERPENOID BIOSYNTHESIS
Terpenoid backbone biosynthesis
Geranyl-PP
(+)-Bornyl-diphosphate
Comphene
5.5.1.8
4.2.3.-
3.1.7.3
4.2.3.25
4.2.3.15
4.2.3.26
4.2.3.16
4.2.3.20
4.2.3.119
4.2.3.120
4.2.3.10
4.2.3.11
4.2.3.108
4.2.3.111
Geraniol
Geraniol degradation
5.4.4.4
(+)-Linalool
4.2.1.127
Myrcene
(-)-Linalool
(-)-(S)-Limonene
(+)-(R)-Limonene
(-)-α-Pinene
(-)-β-Pinene
(-)-endo-Fenchol
Sabinene hydrate
α-Terpineol
1.14.13.152
1.14.13.151
1.14.13.151
8-Hydroxylinalool
1.14.13.151
8-Oxolinalool
10-Hydroxy-geraniol
1.1.1.324
10-Oxogeranial
Iridodial
Iridotrial
7-Deoxy-loganin
7-Deoxy-loganate
1.14.13.74
1.14.13.74
Loganin
2.1.1.50
Loganate
10-Deoxy-geniposidic acid
1.3.3.9
1.3.3.9
Secologanin
2.1.1.50
Secologanate
Geniposidic acid
Asperuloside
Indole alkaloid biosynthesis
1.14.13.47
1.14.13.48
1.14.13.49
(-)-trans-Isopiperitenol
(-)-trans-Carveol
Perillyl alcohol
1.1.1.223
1.1.1.243
(-)-Isopiperi-tenone
(-)-Carvone
1.3.1.82
(+)-cis-Isopulegone
(+)-Pulegone
1.3.1.81
1.14.13.104
1.3.1.81
(+)-Menthofuran
(+)-Isomenthone
(-)-Menthone
1.1.1.207
1.1.1.208
1.14.13.105
(-)-Menthol
(+)-Neomenthol
(4R,7S)-7-Isopropyl-4-methyloxepan-2-one
1.14.13.80
(+)-trans-Carveol
1.1.1.275
(+)-(S)-Carvone
Limonene and pinene degradation
4.2.3.108
1,8-Cineole
1.14.13.156
6-endo-Hydroxycineole
1.1.1.241
6-Oxocineole
1.14.13.51
1,6,6-Trimethyl-2,7-dioxabicyclo-[3.2.2]nonan-3-one
spontaneous
4,5-Dihydro-5,5-dimethyl-4-(3-oxobutyl)furan-2(3H)-onehexanoate
00902 3/13/14
(c) Kanehisa Laboratories

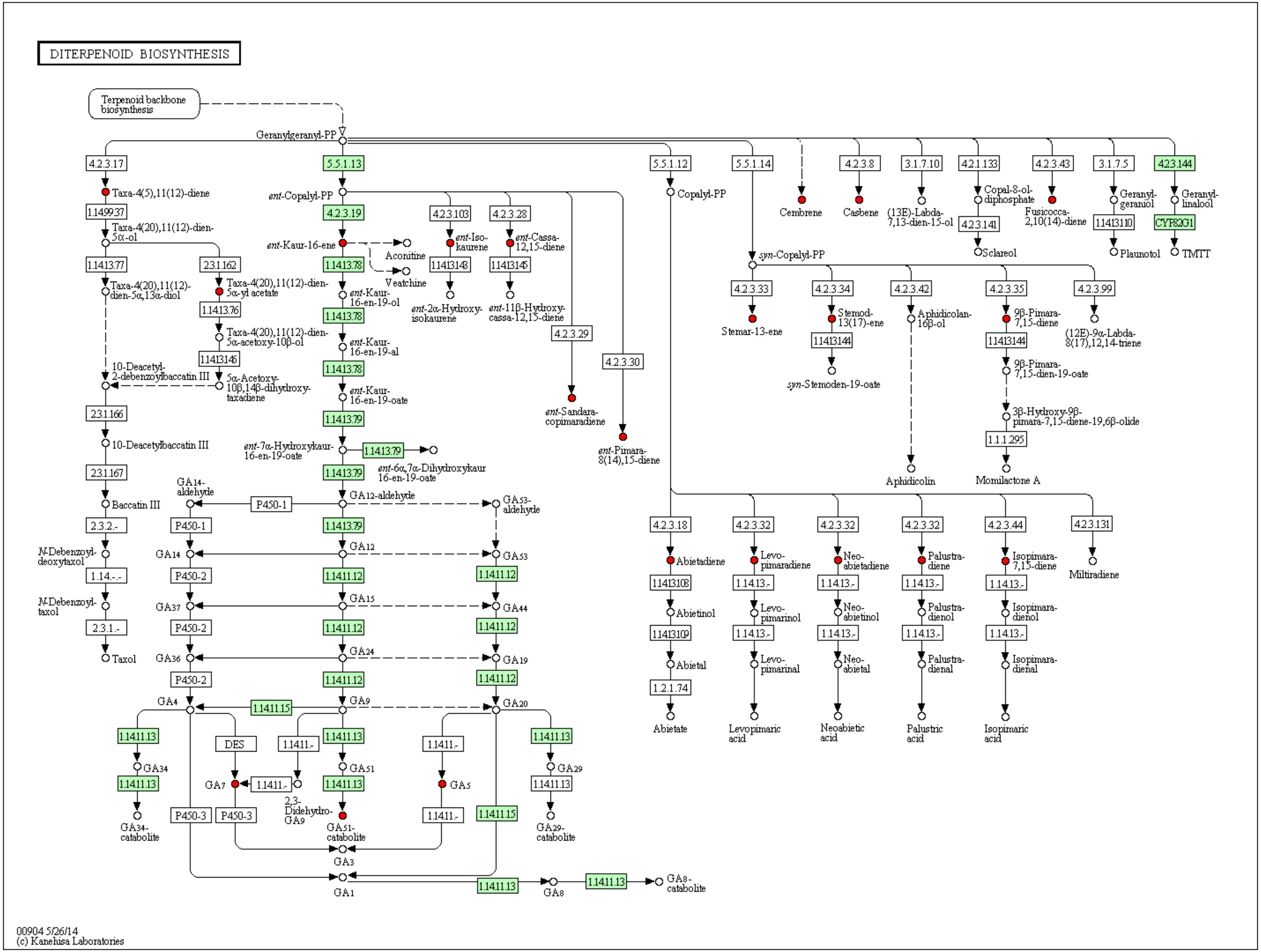
DITERPENOID BIOSYNTHESIS
Terpenoid backbone biosynthesis
Geranylgeranyl-PP
4.2.3.17
Taxa-4(5),11(12)-diene
1.14.99.37
Taxa-4(20),11(12)-dien-5α-ol
1.14.13.77
2.3.1.162
Taxa-4(20),11(12)-dien-5α,13α-diol
Taxa-4(20),11(12)-dien-5α-yl acetate
1.14.13.76
Taxa-4(20),11(12)-dien-5α-acetoxy-10β-ol
1.14.13.146
10-Deacetyl-2-debenzoylbaccatin III
5α-Acetoxy-10β,14β-dihydroxy-taxadiene
2.3.1.166
10-Deacetylbaccatin III
2.3.1.167
Baccatin III
2.3.2.-
N-Debenzoyl-deoxytaxol
1.14.-.-
N-Debenzoyl-taxol
2.3.1.-
Taxol
5.5.1.13
ent-Copalyl-PP
4.2.3.19
ent-Kaur-16-ene
Aconitine
Veatchine
1.14.13.78
ent-Kaur-16-en-19-ol
ent-Kaur-16-en-19-al
ent-Kaur-16-en-19-oate
1.14.13.79
ent-7α-Hydroxykaur-16-en-19-oate
ent-6α,7α-Dihydroxykaur 16-en-19-oate
GA12-aldehyde
GA14-aldehyde
P450-1
GA53-aldehyde
GA12
GA14
GA53
1.14.11.12
P450-2
GA15
GA37
GA44
GA24
GA36
GA19
GA9
GA4
GA20
1.14.11.15
1.14.11.13
GA34
DES
1.14.11.-
GA51
GA7
GA5
GA29
2,3-Didehydro-GA9
GA34-catabolite
P450-3
GA51-catabolite
GA29-catabolite
GA3
GA1
GA8
GA8-catabolite
4.2.3.103
ent-Iso-kaurene
1.14.13.143
ent-2α-Hydroxy-isokaurene
4.2.3.28
ent-Cassa-12,15-diene
1.14.13.145
ent-11β-Hydroxy-cassa-12,15-diene
4.2.3.29
ent-Sandara-copimaradiene
4.2.3.30
ent-Pimara-8(14),15-diene
5.5.1.12
Copalyl-PP
5.5.1.14
syn-Copalyl-PP
Cembrene
4.2.3.8
Casbene
3.1.7.10
(13E)-Labda-7,13-dien-15-ol
4.2.1.133
Copal-8-ol-diphosphate
4.2.3.141
Sclareol
4.2.3.43
Fusicocca-2,10(14)-diene
3.1.7.5
Geranyl-geraniol
1.14.13.110
Plaunotol
4.2.3.144
Geranyl-linalool
CYP82G1
TMTT
4.2.3.33
Stemar-13-ene
4.2.3.34
Stemod-13(17)-ene
1.14.13.144
syn-Stemoden-19-oate
4.2.3.42
Aphidicolan-16β-ol
Aphidicolin
4.2.3.35
9β-Pimara-7,15-diene
9β-Pimara-7,15-dien-19-oate
3β-Hydroxy-9β-pimara-7,15-diene-19,6β-olide
1.1.1.295
Momilactone A
4.2.3.99
(12E)-9α-Labda-8(17),12,14-triene
4.2.3.18
Abietadiene
1.14.13.108
Abietinol
1.14.13.109
Abietal
1.2.1.74
Abietate
4.2.3.32
Levo-pimaradiene
1.14.13.-
Levo-pimarinol
Levo-pimarinal
Levopimaric acid
Neo-abietadiene
Neo-abietinol
Neo-abietal
Neoabietic acid
Palustra-diene
Palustra-dienol
Palustra-dienal
Palustric acid
4.2.3.44
Isopimara-7,15-diene
Isopimara-dienol
Isopimara-dienal
Isopimaric acid
4.2.3.131
Miltiradiene
00904 5/26/14
(c) Kanehisa Laboratories

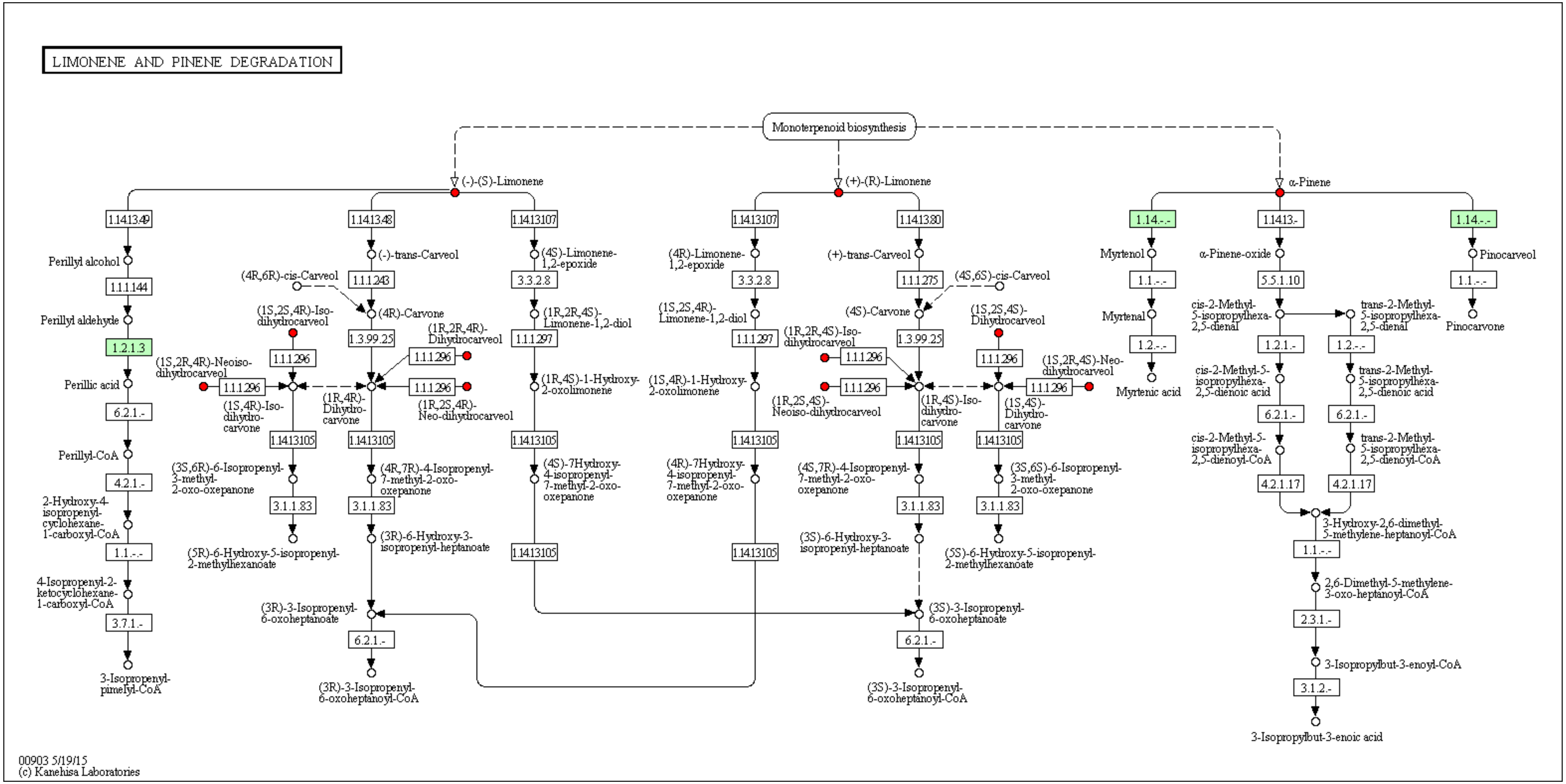
LIMONENE AND PINENE DEGRADATION
Monoterpenoid biosynthesis
(-)-(S)-Limonene
(+)-(R)-Limonene
α-Pinene
1.14.13.49
1.14.13.48
1.14.13.107
1.14.13.107
1.14.13.80
1.14.-.-
1.14.13.-
1.14.-.-
Perillyl alcohol
1.1.1.144
Perillyl aldehyde
1.2.1.3
Perillic acid
6.2.1.-
Perillyl-CoA
4.2.1.-
2-Hydroxy-4-isopropenyl-cyclohexane-1-carboxyl-CoA
1.1.-.-
4-Isopropenyl-2-ketocyclohexane-1-carboxyl-CoA
3.7.1.-
3-Isopropenyl-pimelyl-CoA
(-)-trans-Carveol
(4R,6R)-cis-Carveol
1.1.1.243
(4R)-Carvone
1.3.99.25
(1S,2S,4R)-Iso-dihydrocarveol
(1R,2R,4R)-Dihydrocarveol
(1S,2R,4R)-Neoiso-dihydrocarveol
1.1.1.296
(1S,4R)-Iso-dihydro-carvone
(1R,4R)-Dihydro-carvone
(1R,2S,4R)-Neo-dihydrocarveol
1.14.13.105
(3S,6R)-6-Isopropenyl-3-methyl-2-oxo-oxepanone
(4R,7R)-4-Isopropenyl-7-methyl-2-oxo-oxepanone
3.1.1.83
(5R)-6-Hydroxy-5-isopropenyl-2-methylhexanoate
(3R)-6-Hydroxy-3-isopropenyl-heptanoate
(3R)-3-Isopropenyl-6-oxoheptanoate
6.2.1.-
(3R)-3-Isopropenyl-6-oxoheptanoyl-CoA
(4S)-Limonene-1,2-epoxide
3.3.2.8
(1R,2R,4S)-Limonene-1,2-diol
1.1.1.297
(1R,4S)-1-Hydroxy-2-oxolimonene
(4S)-7Hydroxy-4-isopropenyl-7-methyl-2-oxo-oxepanone
(4R)-Limonene-1,2-epoxide
(1S,2S,4R)-Limonene-1,2-diol
(1S,4R)-1-Hydroxy-2-oxolimonene
(4R)-7Hydroxy-4-isopropenyl-7-methyl-2-oxo-oxepanone
(+)-trans-Carveol
1.1.1.275
(4S,6S)-cis-Carveol
(4S)-Carvone
(1R,2R,4S)-Iso-dihydrocarveol
(1S,2S,4S)-Dihydrocarveol
(1S,2R,4S)-Neo-dihydrocarveol
(1R,2S,4S)-Neoiso-dihydrocarveol
(1R,4S)-Iso-dihydro-carvone
(1S,4S)-Dihydro-carvone
(4S,7R)-4-Isopropenyl-7-methyl-2-oxo-oxepanone
(3S,6S)-6-Isopropenyl-3-methyl-2-oxo-oxepanone
(3S)-6-Hydroxy-3-isopropenyl-heptanoate
(5S)-6-Hydroxy-5-isopropenyl-2-methylhexanoate
(3S)-3-Isopropenyl-6-oxoheptanoate
(3S)-3-Isopropenyl-6-oxoheptanoyl-CoA
Myrtenol
1.1.-.-
Myrtenal
1.2.-.-
Myrtenic acid
α-Pinene-oxide
5.5.1.10
cis-2-Methyl-5-isopropylhexa-2,5-dienal
trans-2-Methyl-5-isopropylhexa-2,5-dienal
1.2.1.-
1.2.-.-
cis-2-Methyl-5-isopropylhexa-2,5-dienoic acid
trans-2-Methyl-5-isopropylhexa-2,5-dienoic acid
6.2.1.-
cis-2-Methyl-5-isopropylhexa-2,5-dienoyl-CoA
trans-2-Methyl-5-isopropylhexa-2,5-dienoyl-CoA
4.2.1.17
3-Hydroxy-2,6-dimethyl-5-methylene-heptanoyl-CoA
1.1.-.-
2,6-Dimethyl-5-methylene-3-oxo-heptanoyl-CoA
2.3.1.-
3-Isopropylbut-3-enoyl-CoA
3.1.2.-
3-Isopropylbut-3-enoic acid
Pinocarveol
1.1.-.-
Pinocarvone
00903 5/19/15
(c) Kanehisa Laboratories

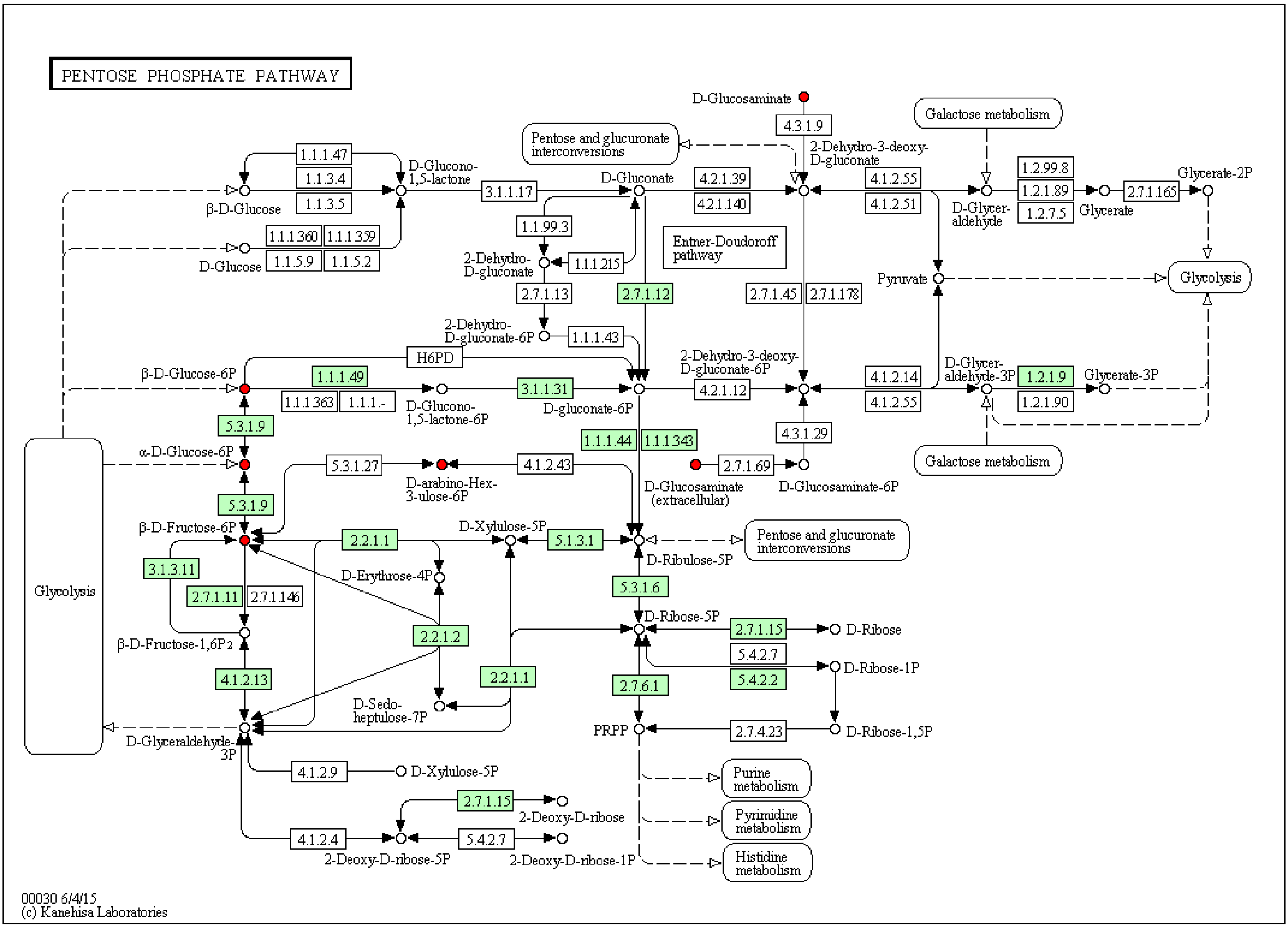
PENTOSE PHOSPHATE PATHWAY
D-Glucosaminate
4.3.1.9
Galactose metabolism
Pentose and glucuronate interconversions
2-Dehydro-3-deoxy-D-gluconate
1.1.1.47
1.1.3.4
1.1.3.5
D-Glucono-1,5-lactone
β-D-Glucose
3.1.1.17
D-Gluconate
4.2.1.39
4.2.1.140
4.1.2.55
4.1.2.51
1.2.99.8
1.2.1.89
1.2.7.5
Glycerate
2.7.1.165
Glycerate-2P
D-Glyceraldehyde
1.1.1.360
1.1.1.359
1.1.5.9
1.1.5.2
D-Glucose
1.1.99.3
2-Dehydro-D-gluconate
1.1.1.215
Entner-Doudoroff pathway
Pyruvate
Glycolysis
2.7.1.13
2.7.1.12
2.7.1.45
2.7.1.178
2-Dehydro-D-gluconate-6P
1.1.1.43
H6PD
2-Dehydro-3-deoxy-D-gluconate-6P
4.1.2.14
4.1.2.55
D-Glyceraldehyde-3P
1.2.1.9
1.2.1.90
Glycerate-3P
β-D-Glucose-6P
1.1.1.49
1.1.1.363
1.1.1.-
D-Glucono-1,5-lactone-6P
3.1.1.31
D-gluconate-6P
4.2.1.12
5.3.1.9
1.1.1.44
1.1.1.343
4.3.1.29
α-D-Glucose-6P
5.3.1.27
4.1.2.43
D-arabino-Hex-3-ulose-6P
2.7.1.69
D-Glucosaminate (extracellular)
D-Glucosamine-6P
Galactose metabolism
5.3.1.9
β-D-Fructose-6P
2.2.1.1
D-Xylulose-5P
5.1.3.1
D-Ribulose-5P
Pentose and glucuronate interconversions
3.1.3.11
D-Erythrose-4P
2.7.1.11
2.7.1.146
5.3.1.6
Glycolysis
β-D-Fructose-1,6P2
2.2.1.2
D-Ribose-5P
2.7.1.15
D-Ribose
5.4.2.7
4.1.2.13
2.2.1.1
2.7.6.1
5.4.2.2
D-Ribose-1P
D-Sedo-heptulose-7P
D-Glyceraldehyde-3P
PRPP
2.7.4.23
D-Ribose-1,5P
4.1.2.9
D-Xylulose-5P
Purine metabolism
2.7.1.15
2-Deoxy-D-ribose
Pyrimidine metabolism
4.1.2.4
5.4.2.7
2-Deoxy-D-ribose-5P
2-Deoxy-D-ribose-1P
Histidine metabolism
00030 6/4/15
(c) Kanehisa Laboratories

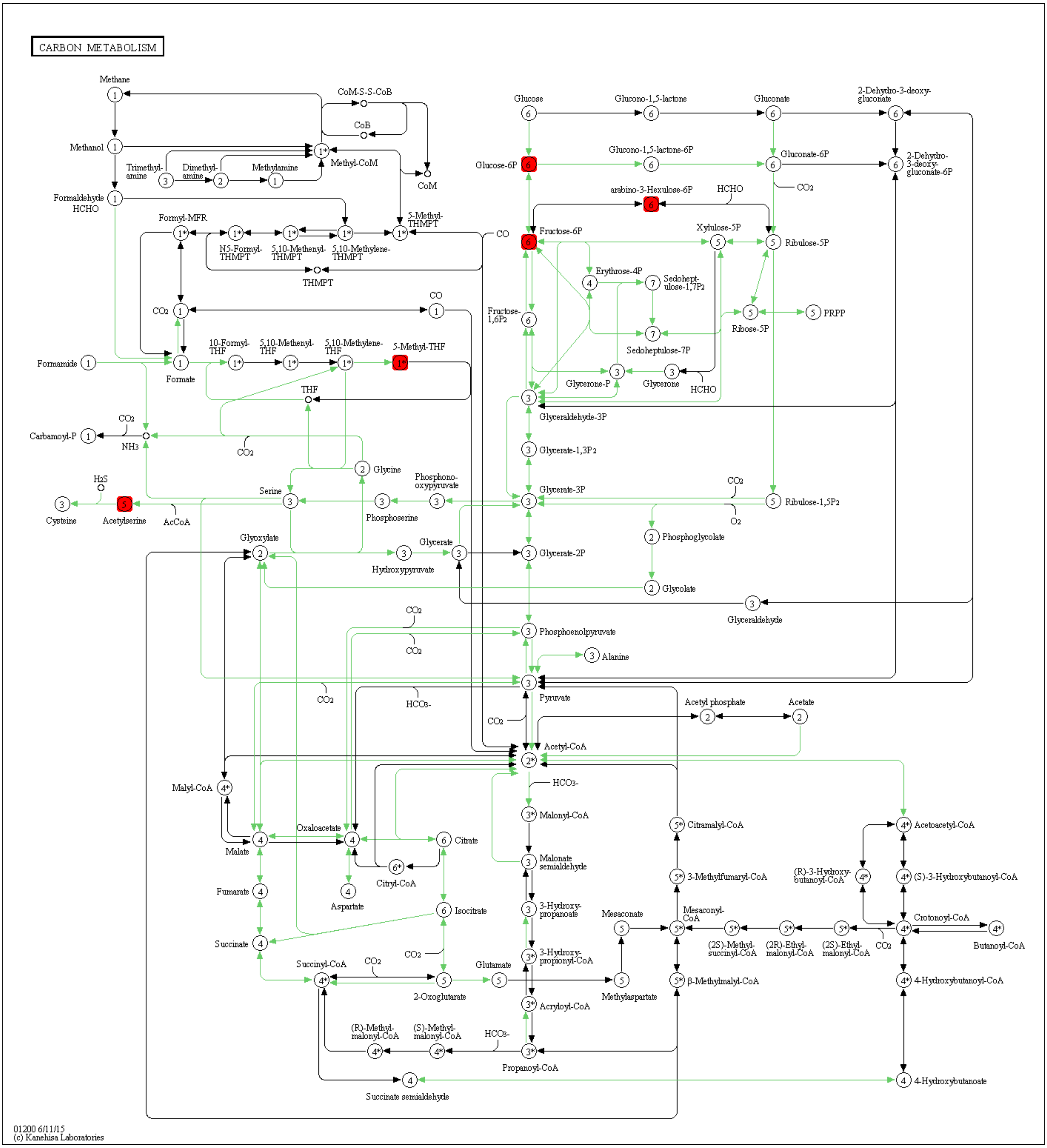
CARBON METABOLISM
Methane
CoM-S-S-CoB
CoB
Methanol
Trimethyl-amine
Dimethyl-amine
Methylamine
Methyl-CoM
CoM
Formaldehyde HCHO
Formyl-MFR
N5-Formyl-THMPT
5,10-Methenyl-THMPT
5,10-Methylene-THMPT
5-Methyl-THMPT
CO
THMPT
CO2
CO
Formamide
Formate
10-Formyl-THF
5,10-Methenyl-THF
5,10-Methylene-THF
5-Methyl-THF
THF
CO2
Carbamoyl-P
NH3
CO2
H2S
Cysteine
Acetylserine
AcCoA
Serine
Glycine
Phosphoserine
Phospho-oxypyruvate
Glyoxylate
Hydroxypyruvate
Glycerate
Glucose
Glucono-1,5-lactone
Gluconate
2-Dehydro-3-deoxy-gluconate
Glucose-6P
Glucono-1,5-lactone-6P
Gluconate-6P
2-Dehydro-3-deoxy-gluconate-6P
arabino-3-Hexulose-6P
HCHO
CO2
Fructose-6P
Xylulose-5P
Ribulose-5P
Erythrose-4P
Sedohept-ulose-1,7P2
Fructose-1,6P2
Ribose-5P
PRPP
Sedoheptulose-7P
Glycerone-P
Glycerone
HCHO
Glyceraldehyde-3P
Glycerate-1,3P2
Glycerate-3P
CO2
Ribulose-1,5P2
O2
Phosphoglycolate
Glycerate-2P
Glycolate
Glyceraldehyde
CO2
Phosphoenolpyruvate
CO2
Alanine
CO2
HCO3-
Pyruvate
Acetyl phosphate
Acetate
CO2
Acetyl-CoA
Malyl-CoA
HCO3-
Malonyl-CoA
Oxaloacetate
Citrate
Malate
Citryl-CoA
Malonate semialdehyde
Citramalyl-CoA
Acetoacetyl-CoA
3-Methylfumaryl-CoA
(R)-3-Hydroxy-butanoyl-CoA
(S)-3-Hydroxybutanoyl-CoA
Fumarate
Aspartate
Isocitrate
3-Hydroxy-propanoate
Mesaconate
Mesaconyl-CoA
Crotonoyl-CoA
Succinate
CO2
3-Hydroxy-propionyl-CoA
(2S)-Methyl-succinyl-CoA
(2R)-Ethyl-malonyl-CoA
(2S)-Ethyl-malonyl-CoA
CO2
Butanoyl-CoA
Succinyl-CoA
CO2
Glutamate
2-Oxoglutarate
Methylaspartate
β-Methylmalyl-CoA
4-Hydroxybutanoyl-CoA
Acryloyl-CoA
(R)-Methyl-malonyl-CoA
(S)-Methyl-malonyl-CoA
HCO3-
Propanoyl-CoA
Succinate semialdehyde
4-Hydroxybutanoate
01200 6/11/15
(c) Kanehisa Laboratories
